# Point contacts in halide perovskite solar cells: from reduced interfacial recombination to increased ionic field screening†

**DOI:** 10.1039/d5el00110b

**Published:** 2025-07-26

**Authors:** Guorui He, Andrés-Felipe Castro-Méndez, Jonas Diekmann, Guus J. W. Aalbers, Paria Forozi Sowmeeh, Arpana Singh, Simon V. Quiroz Monnens, Francisco Peña-Camargo, Martin Stolterfoht, Bernd Stannowski, Heinz Christoph Neitzert, René A. J. Janssen, Christian Michael Wolff, Dieter Neher, Felix Lang

**Affiliations:** a Soft Matter Physics and Optoelectronics, Institute of Physics and Astronomy, University of Potsdam Karl-Liebknecht-Str. 24–25 14476 Potsdam-Golm Germany felix.lang.1@uni-potsdam.de andres.castro@uni-potsdam.de; b École Polytechnique Fédérale de Lausanne (EPFL), Institute of Electrical and Microengineering (IEM), Photovoltaics and Thin Film Electronics Laboratory (PV-Lab) Rue de la Maladière 71b 2000 Neuchâtel Switzerland; c Molecular Materials and Nanosystems & Institute for Complex Molecular Systems, Eindhoven University of Technology P. O. Box 513 5600 MB Eindhoven The Netherlands; d Dutch Institute for Fundamental Energy Research De Zaale 20 5612 AJ Eindhoven The Netherlands; e Department of Industrial Engineering (DIIN), University of Salerno 84084 Fisciano Italy; f Helmholtz-Zentrum Berlin für Materialien und Energie, Solar Energy Division 12489 Berlin Germany; g Electronic Engineering Department, The Chinese University of Hong Kong Sha Tin N. T. Hong Kong SAR China; h PVcomB, Helmholtz-Zentrum Berlin 12489 Berlin Germany; i Beuth University of Applied Sciences Berlin 13353 Berlin Germany

## Abstract

The performance of p–i–n structured perovskite solar cells (PSCs) is primarily limited by the charge recombination at the interface between the perovskite and the electron transporting layer, most commonly C_60_. Inspired by the silicon passivated emitter rear cell design, we propose point contacts (PCs) to reduce the recombination at the perovskite/C_60_ interface. Inserting PCs between the perovskite and C_60_ layers enables an increased efficiency from 18.9% to 20.0%, which mainly originates from the reduced non-radiative recombination that leads to a higher open-circuit voltage (*V*_OC_) from 1.16 to 1.21 V. Combining a lithium fluoride (LiF) layer beneath the PCs (perovskite/LiF/PCs) can further boost the *V*_OC_ to 1.26 V, reaching 90% of the detailed balance limit. However, we find that PCs exacerbate the effect of mobile ions in PSCs, accelerating the degradation under *operando* conditions. Our results reveal that mobile ions accumulate at the PCs, triggering a faster degradation of the device. These observations are further supported by one- and two-dimensional drift-diffusion simulations that confirm the accumulation of ions at the PCs. This work, therefore, highlights the importance of ion management for improved stability and points to a new degradation mechanism when a discontinuous insulating layer forms at the perovskite interfaces.

## Abstract 2

Inserting point contacts between the perovskite and C_60_ layers reduces the interfacial recombination. However, the accumulation of mobile ions at the point contacts can decrease the device stability.

## Graphical abstract

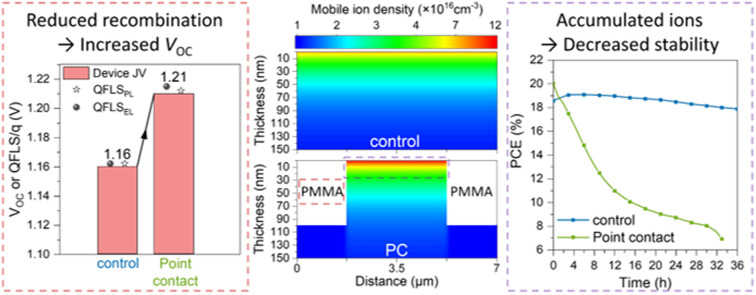

**Broader context**

Perovskite solar cells (PSCs) and perovskite/silicon solar cells have reached impressive efficiencies. Today, their efficiency is often limited by parasitic surface recombination, especially at the perovskite/C60 interface. While many passivation materials are explored, a powerful, yet barely used technique to reduce interfacial recombination is the utilization of point contacts (PCs). PCs are inspired by the silicon (Si) passivated emitter and rear cell (PERC) technology, where an insulating dielectric layer with determined openings is placed at the interface between the absorber material and the back metal contacts. In this approach, charges can be extracted to the selective electrodes only at the formed local openings, reducing the area where charges recombine parasitically, thus increasing the open-circuit voltage (*V*_OC_) and efficiency. We demonstrate that PCs lead to an increase in the power conversion efficiency in PSCs. Our optimized devices suppress interfacial recombination and reach a *V*_OC_ of up to 1.26 V, resembling 90% of the detailed balance limit, with improved performance. However, PCs accelerate the degradation under *operando* conditions, pointing towards a novel degradation mechanism. Our work thus reveals a new degradation mechanism for PSCs utilizing PCs and emphasizes the importance of ion management to enable future use of point contact strategies. Moreover, the exact degradation mechanism can be applied to any discontinuous passivation layer with insulating properties, where PCs are formed unintentionally.

## Introduction

Organic–inorganic hybrid halide perovskite solar cells (PSCs) have attracted worldwide attention as one of the most promising technologies for photovoltaic (PV) applications, due to their excellent intrinsic optoelectronic properties,^1^ such as low exciton binding energy,^2^ long carrier diffusion length^3^ and high absorption coefficient.^4^ Recently, the state-of-the-art PSCs have achieved a record power conversion efficiency (PCE) of 26.7% and combining a silicon solar cell to fabricate silicon/perovskite tandem solar cells can further increase the PCE to 34.2%.^5^ However, compared to silicon solar cells, PSCs still suffer from defects, such as vacancies, interstitials, grain boundaries, dangling bonds, and undercoordinated Pb^2+^, which increase the non-radiative recombination at the interfaces and limit the device performance.^6–10^ Although these defects are also present in the bulk, they are especially detrimental at the interface between the perovskite/transporting layer (TL) interfaces, where they create traps, particularly when paired with C_60_ in the p–i–n configuration.^11–14^

So far, different defect passivation methods have been proposed to increase the efficiency and stability of the PSCs, including ammonium salts,^15,16^ thin layer passivation,^17–19^ Lewis acids and bases,^20,21^ among others. Solution-processed thin polymer layer passivation has shown to be effective with different polymers, including polystyrene, Teflon and fluoro-silane,^17^ in combination with various perovskite compositions.^18^ Different passivation mechanisms have been proposed for the thin layer passivation, such as contact displacement,^22,23^ chemical passivation *via* the functional groups,^18^ grain boundary filling^19,24^ and charge tunneling.^17^ Polymethyl methacrylate (PMMA), as a Lewis base,^18^ has been widely used as a thin-passivation layer in PSCs, to improve the crystallization of the perovskite as a nucleation template,^25^ and to passivate the defects between the perovskite and TLs.^18,24,26,27^ To mitigate the insulating effects of PMMA, phenyl-C61-butyric acid methyl ester (PCBM) has been combined with PMMA.^28–30^ A different approach to reduce interfacial recombination is the fabrication of point contacts (PCs). PCs are inspired by the silicon passivated emitter and rear cell (PERC) technology, where an insulating dielectric layer with determined openings is placed at the interface between the absorber material and the back metal contacts.^31^ In this approach, charges can be extracted to the selective electrodes only at the formed local openings, reducing the area where charges can be trapped and recombine, thus increasing the open-circuit voltage (*V*_OC_) and short-circuit current density (*J*_SC_). However, when the improvement in the pseudo-fill-factor stemming from less recombination is outweighed by the higher resistances caused by lateral bulk current flow, the fill factor (FF) can decrease.^32^

Opening the insulating dielectric layer in PERC-based silicon solar cells requires high-power lasers, which cannot be transferred to PSCs because of their sensitivity to localized heat. Therefore, many works implemented PCs before deposition of the perovskite, *e.g.* Vomer–Weber growth of isolated Al_2_O_3_ islands or TiO_2_ nanorods at the bottom interface.^26,33^ For p–i–n based PSCs, where recombination at the top perovskite/C_60_ interface is crucial, patterned lithium fluoride (LiF) evaporated through a shadow mask has recently been implemented by Mao *et al.*, achieving a certified PCE of 24.95%.^34^

**Fig. 1:**
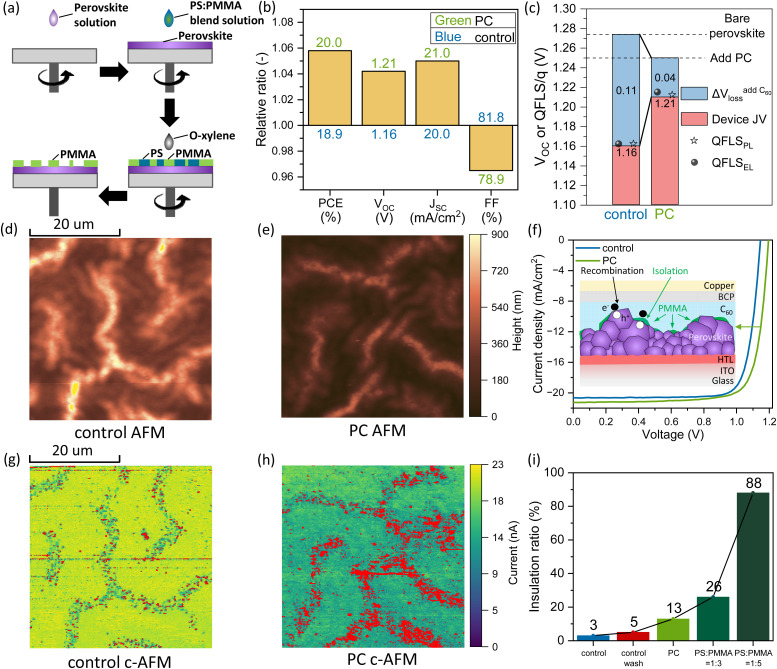
(a) Schematic of the PC formation process. (b) A direct comparison of the photovoltaic parameters between the “control” and PC device in relative ratio and absolute value (see Fig. S4 for statistics†). (c) Summary of the QFLS calculated from PLQY (QFLS_PL_) of films and devices, QFLS calculated from ELQY (QFLS_EL_) of devices and the *V*_OC_ of the devices. AFM images of (d) perovskite/C_60_ and (e) perovskite/PC/C_60_ films with the same length scale and scale bar. (f) Schematic of the PC device structure as well as the *J*–*V* characteristics of the “control” and PC device under AM 1.5G and 100 mW cm^−2^ illumination. c-AFM images of (g) perovskite/C_60_ film and (h) perovskite/PC/C_60_ film with −10 V bias from the tip and same scale bar. The red color highlights the areas where the extracted current is less than 50% of the maximum current. (i) The “insulation ratios” extracted from c-AFM on films in different conditions with a C_60_ layer on top.

In this study, we used a blend solution of polystyrene (PS) and PMMA combined with a selectively PS-dissolving solvent to fabricate PCs at the perovskite/C_60_ interface, aiming to explore the working mechanisms of PCs in PSCs. Different from the thin polymer layer passivation mentioned above,^17–19,24^ we deposited a high-concentration (25 mg mL^−1^) blend polymer solution onto the perovskite layer, forming a compact insulating layer with a thickness of ∼70 nm. Subsequently, the PS in this layer is removed *via* washing with *ortho*-xylene (*o*-xylene), which results in a discontinuous PMMA layer (see Fig. 1a). The optimal device performance is achieved with a PS : PMMA weight ratio of 1 : 2. This enhances the PCE from 18.9% to 20.0% compared to the “control” devices. The improvement is mainly due to an increase in the *V*_OC_ of 50 mV. However, while the initial device performance is enhanced, ions accumulate at these open contacts under *operando* conditions, triggering a significant degradation pathway that decreases the long-term stability of the device. Our two-dimensional drift-diffusion simulation demonstrates that lowering mobile ion density in PSCs with PCs can further boost *V*_OC_ and FF, without affecting the long-term stability.

## Results and discussion

### Improvement of photovoltaic performance and characterization of optoelectronic properties

To investigate the PC strategy, we introduced a discontinuous polymer between perovskite and C_60_ in the device structure: glass/ITO/CbzNaph/perovskite/PC/C_60_/BCP/Cu. Here, ITO stands for indium tin oxide; CbzNaph is (4-(7*H*-dibenzo[*c*,*g*]carbazol-7-yl)butyl)phosphonic acid; the perovskite composition is Cs_0.21_FA_0.74_MA_0.05_PbI_0.81_Br_0.14_Cl_0.05_ and BCP is bathocuproine. As determined from the peak of the external quantum efficiency (EQE_PV_) derivative of the devices (see Fig. S2 in the ESI†), the perovskite has a bandgap of 1.68 eV, which is ideal for silicon/perovskite tandem application.^35^ The discontinuous polymer layer was created by depositing a high concentration (25 mg mL^−1^) blend solution of PS and PMMA onto the perovskite layer, forming a ∼70 nm compact polymer insulating layer (Fig. S3†). Subsequently, the blend polymer is washed with a selectively PS-dissolving solvent (*o*-xylene), which dissolves PS and leaves a discontinuous PMMA layer. Details on the fabrication process are described in the Experimental section in the ESI.† To optimize the PCE of the PSCs with PCs, the weight ratio of the PS and PMMA solution was varied from 1 : 0.5, over 1 : 1, 1 : 2, 1 : 3 to 1 : 5. To compare the PC strategy with the thin layer passivation, we also included devices with a thin PS or PMMA layer (1 mg mL^−1^ in concentration) between the perovskite and C_60_ (Fig. S4†). It is important to note that in this work, films and devices without a polymer layer are denoted as “control”, and films and devices that include the *o*-xylene treatment after the perovskite layer, but not the polymer, are denoted as “control wash”.

On the one hand, we identify that the washing with the *o*-xylene, does not significantly influence the performance of devices, leading to identical *J*–*V* characteristics to “control” devices with *V*_OC_ of ∼1.16 V, *J*_SC_ of ∼20.0 mA cm^−2^, FF of ∼81.8% and PCE of ∼19.0%. This benign effect of the *o*-xylene wash is further confirmed by the photoluminescence quantum yield (PLQY) values of devices (Fig. S5 and S6†). On the other hand, the formation of PCs on the perovskite increases the *V*_OC_, while decreasing the FF, especially at higher weight ratios of PMMA. When the ratio of PS : PMMA reaches 1 : 5, all device parameters drop vastly, indicating large extraction losses caused by a thick insulating layer without enough open contacts (Fig. S7†). The optimal device performance is achieved with a PS : PMMA ratio of 1 : 2, and the device based on this ratio is denoted as PC device in the following discussion. The performance parameters are: *V*_OC_ of 1.21 V, *J*_SC_ of 21.0 mA cm^−2^, FF of 78.9% and PCE of 20.0%. Compared to the “control” device, *J*_SC_ slightly increases by 1 mA cm^−2^; *V*_OC_ is enhanced by 50 mV, and FF decreases by 2.9%. The relative ratios and absolute values of the photovoltaic parameters are summarized in Fig. 1b. The trade-off between increasing *V*_OC_ and decreasing FF with a lower area fraction of PCs is predicted by simulation results from G. D. Tabi *et al.*^36^ We note that a thin PS or PMMA passivation results in a *V*_OC_ increase of 30 mV, which is lower compared to our PC strategy yielding 50 mV.

To investigate the origin of the *V*_OC_ increase in the PC device, we measured the PLQYs on bare perovskite film and complete devices with or without PCs on top of the perovskite layer and extracted the quasi-Fermi-level splittings (QFLS_PL_s), see ESI Note 1 and Table S1 in the ESI.† The bare perovskite film exhibits a QFLS_PL_ of 1.27 eV (PLQY of 1.55 × 10^−2^). However, for the complete device structure, the direct contact of perovskite with the C_60_ layer lowers the QFLS_PL_ from 1.27 to 1.16 eV, corresponding to a ∼80-fold decrease in PLQY. In comparison, by incorporating PCs, the presence of C_60_ layer only lowers the QFLS_PL_ of the full device stack from 1.25 eV to 1.22 eV (from PLQY of 6.46 × 10^−3^ to 1.55 × 10^−3^), indicating that PCs effectively reduce recombination at this interface. Furthermore, we measured the electroluminescence quantum yields (ELQYs) of the “control” and PC devices, and obtained the quasi-Fermi-level splittings (QFLS_EL_s), which align well with the measured QFLS_PL_s and *V*_OC_ of the devices (ESI Note 1, Fig. S9 in the ESI†).^37–39^ The QFLSs and *V*_OC_ values of the “control” and PC device are summarized in Fig. 1c. The match between QLFSs and *V*_OC_ confirms that the higher *V*_OC_ of the PC device is due to reduced interfacial recombination rather than a better charge transport or energy alignment compared to the “control” device.^23,37,40^ The EQE_PV_ spectra of the “control” and PC devices shown in Fig. S10† reveal a higher photocurrent generation for the PC device and the integrated EQE_PV_ matches the *J*_SC_ well with an error within ± 3%. The increased *J*_SC_ is attributed to a higher reflection at the perovskite/PMMA interface, compared to perovskite/C_60_, which originates from a larger refractive index mismatch between the perovskite and PMMA, see Fig. S11,† and the passivation effect of the PC layer can also contribute.^18^

### Morphological characterization of point contacts

To reveal the surface morphology of perovskite with and without PCs on top, we measured scanning electron microscopy (SEM) utilizing an immersion lens secondary electron (in-lens) detector on “control”, “control wash” and PC perovskite films, see Fig. S12 in the ESI.† The perovskite films exhibit a wrinkled structure, having mountain- and valley-like regions (Fig. S13†), which are attributed to the in-plane compressive stress during crystallization.^41^ SEM images reveal the presence of brighter crystals that accumulate mainly at the “mountains”. These crystals have been linked to lead iodide,^42,43^ which agree well with the XRD patterns of our samples (Fig. S14†). By comparing the SEM images of the “control” and “control wash” films in Fig. S12a and S12b,† respectively, we note that *o*-xylene wash can narrow the “mountains”.^44^ Despite notable changes in the surface morphology with the *o*-xylene wash, XRD patterns of the “control”, “control wash” and PC perovskite film (Fig. S14†) suggest unchanged bulk properties of the perovskite film.^18^ To better resolve the surface topography, a secondary electron detector located to the side (SE detector) was also used. SEM images with in-lens and SE detector at the same spots (Fig. S15†) reveal polymer clusters on PC samples, while such features are not observed in “control” or “control wash” perovskite films. To improve the topological contrast, a thin layer of gold (∼8 nm) was sputtered on top of the films prior to SEM measurements. In this case, with the SE detector, clear perovskite grains are observed in “control” (Fig. S16a†) and “control wash” perovskite films (Fig. S16b†), while the discontinuous PMMA layer of the PC films partially screens the perovskite morphology. At higher PS : PMMA weight ratios, the morphology of the perovskite layer underneath is further concealed, demonstrating higher coverage of the insulating PMMA layer, as shown in Fig. S16d–f.†

While SEM images confirm the presence of residual PMMA on the perovskite surface, the morphology of the PMMA layer cannot be well resolved, as the electron beam easily penetrates the thin polymer, resulting in insufficient contrast. Therefore, we conducted atomic force microscopy (AFM) and conductive atomic force microscopy (c-AFM) measurements on perovskite/C_60_ and perovskite/PC/C_60_ films on ITO substrates.^45–47^ As shown in Fig. 1d and e, both “control” and PC perovskite films with C_60_ on top exhibit the wrinkled structure observed in SEM images. Fig. 1g and h show the conductivity maps at the same spot. In these maps, red color is used to highlight the areas where the extracted current is less than 50% of the maximum current to determine the insulating area. For the “control” sample, a lower conductivity in the mountain-like areas is observed with a negligible amount of insulating spots, which we attribute to the lower conductivity of lead iodide crystals.^42^ In comparison, more insulating areas are formed in the mountain-like regions for the PC sample, generating in between randomly distributed PCs. By dividing the area of insulating spots by the total image area, we obtained the “insulation ratios”, which allows us to quantify the contact fraction of the PCs. Fig. 1i summarizes the “insulation ratios” extracted from c-AFM images of perovskite films with different conditions (Fig. S17†). A comparable “insulation ratio” of 3% and 5% is observed for the “control” and “control wash” perovskite film, respectively, while it increases from 13% for the PC film with a PS : PMMA weight ratio of 1 : 2 (PS : PMMA = 1 : 2) to 26% with PS : PMMA = 1 : 3. The ratio reaches 88% with PS : PMMA = 1 : 5, resulting in a significant resistance, which corroborates with the poor device performance (Fig. S7†). We also note that the PMMA layer not only creates insulating regions but also lowers the overall conductivity of the film. This could indicate the formation of smaller PCs in the valley-like areas that cannot be resolved with the c-AFM or the presence of a very thin polymer layer that increases the series resistance, which still allows the charge extraction.

Using the morphological data, we created schematic illustrations of PSC incorporating PCs on the perovskite surface and plotted the corresponding current density–voltage (*J*–*V*) curves in Fig. 1f. The discontinuous PMMA layer partially covers the surface of the perovskite bulk layer, insulating the perovskite from the C_60_ layer and thus mitigating the non-radiative recombination at the perovskite/C_60_ interface, leading to a *V*_OC_ increase.

### Charge recombination, extraction and decay

**Fig. 2:**
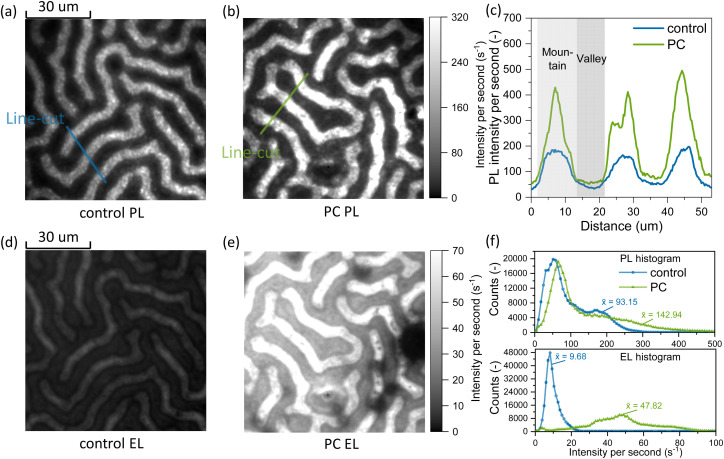
The PL images of (a) “control” and (b) PC device from the glass side with the same length scale and scale bar. (c) Line-cut profiles of the “control” (blue) and PC device (green). See Fig. S20 in the ESI for normalized data.† The EL images of (d) “control” and (e) PC device from the glass side, see Fig. S21† for line-cut profiles from EL images. (f) PL and EL histograms extracted from the corresponding PL and EL images.

To investigate the effects of PCs on the non-radiative recombination in PSCs, we measured photoluminescence (PL) and electroluminescence (EL) images at the same spot from the glass side. The PL images for the “control” and PC device are shown in Fig. 2a and b, respectively. PL and AFM images on the same spots are used to correlate the features in the PL and EL images with the morphology of the films. Independent of illumination from perovskite or glass side, mountain-like regions are found to be more emissive than valley-like in both PL and EL (Fig. S18 and S19†), which is attributed to the charge funneling from higher-bandgap sites to lower-bandgap regions, as previously reported.^43,48,49^Fig. 2c presents line-cut PL profiles across the “mountains” and “valleys” for the “control” (blue line) and the PC device (green line). PCs enhance the PL overall, and a greater increase of PL is observed at the “mountains”, where the PMMA forms more insulating areas, consistent with the results from c-AFM (Fig. 1g and h). Similar “mountains” and “valleys” correlated bright and dark features are observed in EL images as shown in Fig. 2d and e. Different from PL, by normalizing the EL intensity, we observe comparable relative values at the “mountains” and “valleys” for the “control” and PC device (Fig. S21†). This is because the PMMA hinders the charge injection at the “mountains”, and thus more charges recombine at the “valleys”. Fig. 2f presents the PL and EL histograms, demonstrating that PCs enhance PL and EL intensity, which correlates with the calculated PLQY and ELQY values.

Sub-bandgap defects have recently been shown to affect non-radiative loss.^50^ To investigate the effect of PCs on sub-bandgap defect states, we conducted sensitive EQE_PV_ measurements on the “control”, “control wash” and PC devices. As shown in Fig. S24a,† a comparable EQE_PV_ signal and an identical sub-bandgap feature at the photon energy range of 1.10–1.44 eV are observed for the “control” and “control wash” devices. While the EQE_PV_ signal is lowered by the PCs at a photon energy from 1.23 to 1.44 eV, revealing a reduced defect contribution due to mitigated perovskite/C_60_ contact area,^51,52^ an additional defect state peaking at ∼0.93 eV is observed. Sensitive EQE_PV_ measurements on devices with PMMA and PS thin layer passivation in Fig. S24b† show that the defect state at ∼0.93 eV might be due to the electron-trapping nature of the PMMA. Similar defect state at a lower photon energy has also been observed in choline chloride passivated PSCs and does not significantly contribute to non-radiative voltage loss.^51^

**Fig. 3:**
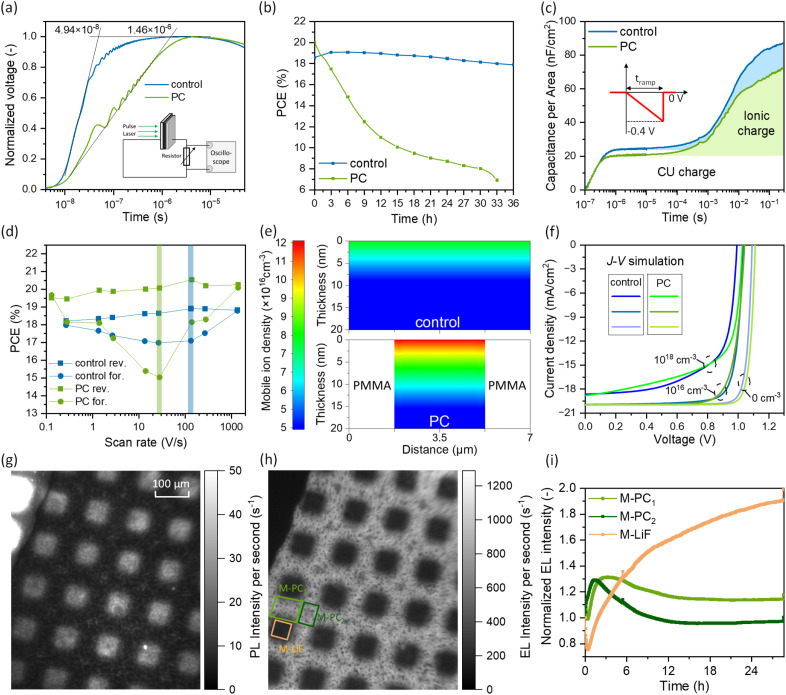
(a) RPV measurements on the “control” and PC device in series connection with a resistance of 100 MΩ and laser pulse with a wavelength of 532 nm. The schematic inset shows the electrical circuit of RPV measurement. (b) SPO stability measurements on the “control” and PC device. (c) Transient signals measured *via* dark-CELIV. The schematic inset shows the voltage ramp from 0 V to −0.4 V within the ramping time, see data of “control wash” device in Fig. S28.† (d) PCE of the “control” and PC device measured with a forward and reverse scan at a scan rate ranging from ∼0.2 to ∼1400 V s^−1^. (e) Simulated distribution of the mobile ions at the first 20 nm thickness of the “control” and PC perovskite layer at steady state under an applied voltage (*V*_app_) of 0.95 V with an overall mobile ion density of 1 × 10^16^ cm^−3^. The mobile ion distribution across the active layer is provided in Fig. S31.† (f) The simulated *J*–*V* curves of the “control” and PC devices with different mobile ion densities based on the geometries shown in Fig. 3e. The (g) PL image and (h) EL image under one-sun-equivalent injection current of M-PC device at the same area from the glass side. (i) The *in situ* EL intensity extracted from the corresponding contours in Fig. 3h with the initial intensity normalized to unity. The *in situ* EL images are provided in Fig. S35 and Video S3.†

To investigate the charge extraction dynamics and understand the effect of PCs on the FF, we conducted resistance-dependent photovoltage (RPV) measurements on the “control” and PC device.^53,54^ In RPV, the transient photocurrent generated by a laser pulse is converted into a voltage by using a high resistance (100 MΩ) and the transient voltage is recorded. Slow collection of electrons and/or holes at the corresponding electrode will be reflected in a slow photovoltage rising time (*t*_r_). *t*_r_ is determined as 4.94 × 10^−8^ s for the “control”, while a dramatically longer *t*_r_ of 1.46 × 10^−6^ s for PC device is observed, as shown in Fig. 3a, which demonstrates that the discontinuous PMMA layer hinders charge extraction, accounting for the lower FF.

To study the charge decay dynamics in the PCs, we conducted time-resolved microwave conductivity (TRMC) measurements on the “control”, “control wash” and PC perovskite films.^55^ In TRMC measurements, the change in the reflected microwave power (Δ*P*/*P*) is induced by the excess charge carriers generated by the pulse laser and is dependent on the charge carrier mobilities.^56^ As shown in Fig. S25,† a higher Δ*P*/*P* value and a decreased decay parameter (*α*) are observed for the PC film compared to the “control” and “control wash”, indicating that the inserted PMMA layer reduces the charge recombination at the perovskite/C_60_ interface, consistent with our PLQY data.

### Effects of mobile ions and degradation mechanisms

The stability of encapsulated devices was tested by tracking the stabilized maximum power output (SPO) under one-sun equivalent illumination at 35 °C. Surprisingly, PCs have a large effect on the stability of the device. The PC device drops to about 35% of the initial performance after 33 h, whereas the “control” device retains more than 95% in the same time, as shown in Fig. 3b. An identical stability test on the “control wash” device (Fig. S26†) can exclude the effects of *o*-xylene washing, pointing to a general instability caused by the PCs. Faster degradation of the PC device is also observed by tracking the maximum power point (MPP) of devices in nitrogen at 25 °C under one-sun equivalent illumination (Fig. S27†).

Since mobile ions have been shown to accelerate the degradation of PSCs,^57–59^ we performed dark-charge extraction *via* linearly increasing voltage (dark-CELIV) and the transient signals are shown in Fig. 3c. In dark-CELIV, the shaded area shown in Fig. 3c is proportional to the ion density, although due to the limitation of the measurement, it would be an underestimation of the ion density.^60,61^ The results indicate that the ion density in the “control” and PC device is comparable, again pointing to unchanged intrinsic bulk properties by PCs. Then, we utilized fast hysteresis (FH) measurements to investigate the impacts of mobile ions on the performance of the PSCs (see PCE at different scan rates in Fig. 3d and *V*_OC_, *J*_SC_, and FF in Fig. S29 in the ESI†).^60,62,63^ The ionic losses, defined as the PCEs difference at low scan and high scan rate, are comparable for the “control” (0.72%) and the PC device (0.69%). Importantly, a larger hysteresis is observed for the PC device and the peak hysteresis position shifts to a lower scan rate of ∼30 V s^−1^, compared to the peak position of ∼130 V s^−1^ for the “control” device, indicating that the PCs cause severe ionic field screening and extend the time for the mobile ions to reach these open contacts.^60^

To further confirm the experimental results, we first simulated the hysteresis behavior of the “control” and PC device by performing a one-dimensional (1-D) drift-diffusion simulation with different scan rates, see Fig. S30 in the ESI† for simulation results and Simulation S1 for simulation details.† We simplified the simulation model by increasing the thickness of the perovskite bulk layer to emulate the longer distances that charge carriers and ions travel in the PC device. Our FH simulation demonstrates that this distance has a strong effect on peak hysteresis, where longer distances cause a shift toward a lower scan rate that mainly originates from the *V*_OC_ and FF. Therefore, this simulation corroborates that the presence of PCs can cause a larger hysteresis peak and the shift to a lower scan as observed in the FH experimental results in Fig. S29.† However, we do not exclude the contribution of slow-moving trapped charges to the hysteresis.^64^

To reveal the mobile ion distribution in the “control” and PC perovskite bulk layer, we utilized a two-dimensional (2-D) drift-diffusion simulation, as described in Simulation S2 in the ESI.† The simulated distribution of the mobile ions in the first 20 nm thickness of the perovskite layers in Fig. 3e exhibits almost twice as high mobile ion density at the perovskite/C_60_ interface (“0 nm thickness” side) in the PC perovskite layer as that in the “control”, with 1.21 × 10^17^ cm^−3^ against 7.75 × 10^16^ cm^−3^. Based on this mobile ion distribution, we simulated *J*–*V* curves of “control” and PC devices with varied mobile ion density (0 cm^−3^, 10^16^ cm^−3^ and 10^18^ cm^−3^) as shown in Fig. 3f, which allows us to quantitively compare the effects of mobile ions on the photovoltaic parameters of “control” and PC devices (Table S2†). Without mobile ions, the formation of PCs increases the *V*_OC_ from 1.09 V to 1.11 V and the FF from 86.9% to 87.4%, resulting in a PCE enhancement from 19.0% to 19.4%. On the other hand, for a mobile ion density of 10^16^ cm^−3^, PCs will decrease the FF from 80.9% to 79.0% without *V*_OC_ gain, leading to a lower PCE. With a further increase of the mobile ion density to 10^18^ cm^−3^, PCs enhance the *V*_OC_ by 40 mV, although the FF would decrease from 66.0% to 63.8%, which enables the PCE of the PC device to again surpass that of “control” device, and the trend of *V*_OC_ increase and FF decrease is consistent with the experimental photovoltaic parameters of “control” and PC devices.

Based on these simulation results, we hypothesize that the areas with the presence of PCs serve as hot spots for degradation, prompted by the increased concentration of ions in these areas. This is consistent with the works by Jacobs *et al.*^58^ that show ions can migrate laterally from outside the active area of a device and accelerate the degradation.

The presence of hot spots for degradation induced by the PCs was confirmed through *in situ* EL imaging. Encapsulated devices were subjected to a five-sun-equivalent injection current to accelerate degradation under the microscope. During this experiment, the formation and rapid propagation of dark spots are observed in the PC device, specifically along the “mountains” where the PMMA forms the PCs. Conversely, in the “control” device, the degradation mechanism differed. It was initiated from random spots across the sample, which grew at a slower rate. These degradation behaviors for the “control” and PC device are illustrated in Fig. S33, Video S1 and S2 in the ESI.† To further prove our hypothesis, we switched to another perovskite composition, Cs_0.05_FA_0.98_MA_0.02_Pb(I_0.98_Br_0.02_)_3_, which does not exhibit wrinkled structures, and used a mesh shadow mask to evaporate LiF (∼5 nm thick), fabricating mesh point contact (M-PC) devices (Fig. S34†). PL and EL images of the M-PC device at the same spot (Fig. 3g and h, respectively) show lower PL intensity and higher EL intensity in the PC regions, consistent with the PL, EL line-cut profiles of PC devices. By measuring *in situ* EL imaging, we again observe faster propagation of dark spots in the M-PC regions (Fig. S35 and Video S3†). To compare the degradation pathways in M-PC and LiF-insulated regions of the M-PC device, we plotted the normalized EL intensity extracted from the three contours shown in Fig. 3h*versus* time (Fig. 3i). At the initial time range, vertical mobile ion movement leads to ion accumulation at the corresponding perovskite/TL interfaces and simultaneously, a lateral field drives the ions from LiF-insulated to M-PC regions,^58^ leading to increasing EL intensity in the M-PC regions and decreasing EL intensity in the LiF-insulated regions. When degradation dominates in the M-PC regions, the EL intensity starts to decrease, while the EL intensity in the LiF-insulated regions keeps increasing due to an applied constant injection current and the faster degradation in the M-PC regions.

These simulation and experimental results, therefore, emphasize the important role of the mobile ion concentration in device performance and stability. Interestingly, the aforementioned work that recently implemented PCs utilizing patterned LiF at both top and bottom interfaces reports higher stability.^34^ In this case, the stability enhancement could, however, stem from strongly improved out-of-plane growth of the perovskite on patterned LiF with reduced bulk defect density.

**Fig. 4:**
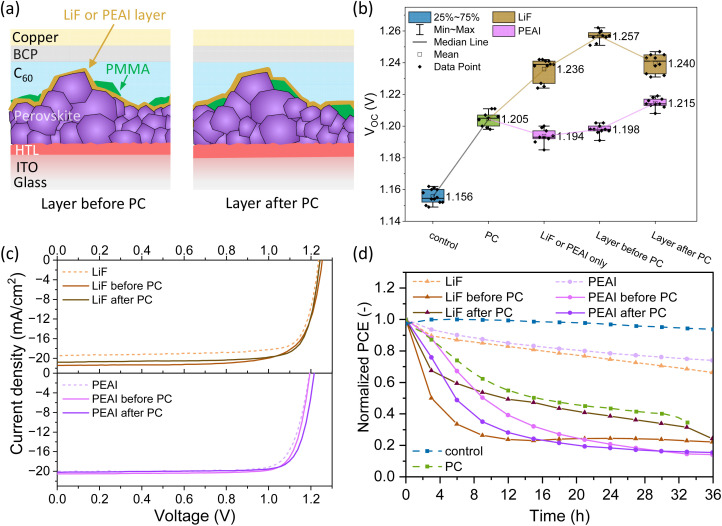
(a) The schematic of the structure with LiF or PEAI layer either before or after the PC deposition. (b) Box diagram showing the *V*_OC_ of the “control”, PC, LiF or PEAI passivated, devices with LiF or PEAI layer either before or after the PCs. (c) The *J*–*V* characteristics of the corresponding devices under AM 1.5G and 100 mW cm^−2^. (d) Normalized PCE of the corresponding devices *versus* time from SPO stability test under one-sun equivalent illumination at 35 °C. “control” and PC devices are added for a direct comparison.

To exclude the possible chemical interaction between perovskite/PMMA or PMMA/C_60_ layer as the main culprit for the induced instability in the device, we separate either the perovskite/PMMA or PMMA/C_60_ layer by inserting other passivation layers: either a layer of ∼0.8 nm LiF *via* thermal evaporation or phenethylammonium iodide (PEAI) *via* solution-processing was deposited either before or after the PC treatment, as shown in Fig. 4a.^65,66^ With a thin LiF passivation layer of ∼0.8 nm, the device exhibits a *V*_OC_ of 1.236 V, while incorporation of the LiF either before or after PC increases the *V*_OC_ to 1.257 and 1.240 V, respectively. Similarly, combining PC with PEAI enhances the *V*_OC_ to 1.198 V for “PEAI before PC” and to 1.215 V for “PEAI after PC”, in comparison to the *V*_OC_ of 1.194 V for PEAI single passivation. The *V*_OC_ improvements are summarized in Fig. 4b. The high *V*_OC_ achieved in “LiF before PC” (1.257 V) reaches ∼90.1% of the detailed balance limit.^67^ However, the FF of the “LiF before PC” and “LiF after PC” devices decreases from 79% to 74% and 78%, respectively, due to the additional insulating LiF layer, while the FF remains around 81% for “PEAI before PC” and “PEAI after PC” under the synergetic effect of insulation from PCs and passivation from PEAI, as shown in Fig. 4c and S36 in the ESI.† Stability test on the devices with either LiF or PEAI passivation layer before or after PC (Fig. 4d) does not show mitigated degradation, indicating that PCs are mainly responsible for the accelerated degradation instead of chemical interactions with PMMA. We also note that LiF and PEAI single layer passivated devices show decreased stability, compared to the “control” device, which was also shown in other literature.^68–70^ The instability has been previously connected to ion-induced degradation.^70–72^ However, we carefully point out that this increase in ionic effect can also be connected to the discontinuities of the more insulating LiF or 2-D PEA_2_PbI_4_ layer, following the same degradation mechanism that we reveal by forming the well-defined PC layer. These results confirm that the ionic nature of metal halide perovskites is a major roadblock to implement PCs in PSCs and indicate the exact degradation mechanism for discontinuous passivation layers with insulating properties. Even though PCs can effectively reduce non-radiative recombination at the perovskite/C_60_ interface, the long-term stability of devices under *operando* conditions is hampered by the creation of hot spots where the ion density increases, prompting the degradation of the device.

## Conclusions

We demonstrated the fabrication of PCs by creating a discontinuous insulating layer of PMMA that partially separates the perovskite and C_60_ layers, where interfacial recombination mainly occurs. This method boosts the *V*_OC_ from 1.16 V to 1.21 V. However, the FF decreases in devices with PCs due to slower charge carrier extraction, as revealed in RPV. The higher *V*_OC_ is corroborated by an increased PLQY and ELQY value, consistent with the TRMC measurements, confirming the suppressed interfacial recombination between the perovskite/C_60_ layer. AFM and c-AFM reveal that the location of PMMA depends on the morphology of the perovskite layer. Having a perovskite layer with wrinkles, exhibiting mountain-like and valley-like areas, allows islands of PMMA to form PCs in between in the mountain-like areas. PL and EL imaging further confirm the insulating effects of the PMMA at the “mountains”. Although PCs effectively reduce interfacial recombination, stability tests show that PCs induce faster degradation under *operando* conditions. While the mobile ion density is comparable for devices with and without PCs from the results of dark-CELIV, a shift of the hysteresis peak to a lower scan rate and a larger hysteresis are observed for devices with PCs from FH measurements. The effect can originate from longer distances ions will travel, which accumulate at the PCs. This accumulation of ions at these spots worsens the ionic field screening and creates hot spots for the degradation of the device. Through *in situ* EL imaging, we observe the formation of these hot spots in the mountain-like regions, where PCs are mainly formed. Similar faster degradation is also observed in the M-PC regions of patterned LiF-insulating devices. The possible chemical interaction between the perovskite/PMMA and PMMA/C_60_ layer as the degradation pathway was excluded by combining PCs with an additional passivation layer, either LiF or PEAI, before or after the PCs. Though the efficiency of the devices is further boosted by combining these passivation and PCs, stability tests show faster degradation for the devices with PCs, and with ∼0.8 nm LiF or PEAI passivation, where PCs might be unintentionally formed. This work highlights that the ionic nature of metal halide perovskite represents a significant challenge to the implementation of PCs in PSCs. Furthermore, the exact degradation mechanism of PCs can be applied to any discontinuous passivation layer with insulating properties.

## Author contributions

G. H. fabricated and characterized the devices and analyzed the data. A. F. C. M. designed the experiments and participated in the device fabrication and characterization. J. D. performed the 2-D drift-diffusion simulation. G. J. W. A. conducted the sensitive EQE_PV_ measurements. P. F. S. performed the 1-D drift-diffusion simulation. A. S. conducted the TRMC measurements. S. V. Q. M. participated in taking the SEM images. F. P. C. participated in PLQY measurements. M. S., B. S., H. C. N., R. A. J. J., C. M. W. and D. N. participated in the data analysis and discussion. F. L. supervised the project. G. H. prepared the manuscript. F. L. and A. F. C. M. contributed to the writing of the paper.

## Conflicts of interest

There are no conflicts to declare.

## Supplementary Material

EL-001-D5EL00110B-s001

EL-001-D5EL00110B-s002

EL-001-D5EL00110B-s003

EL-001-D5EL00110B-s004

## Data Availability

The data that support the findings of this study are available within the article and its ESI.†
