# Supplementary material for: Point contacts in halide perovskite solar cells: from reduced interfacial recombination to increased ionic field screening
Source: EES Solar. 2025 Jul 26;1(5):775–85. doi: 10.1039/d5el00110b (PMC12319528; doi:10.1039/d5el00110b)
Supplement: EL-001-D5EL00110B-s001 [file EL-001-D5EL00110B-s001.pdf]

Supplementary Information (SI) for EES Solar.
This journal is © The Royal Society of Chemistry 2025

Supporting Information (ESI†)

Point Contacts in Halide Perovskite Solar Cells: From Reduced Interfacial Recombination to Increased Ionic Field Screening

Guorui He, Andrés-Felipe Castro-Méndez, Jonas Diekmann, Guus J. W. Aalbers, Paria Forozi Sowmeeh, Arpana Singh, Simon V. Quiroz Monnens, Francisco Peña-Camargo, Martin Stolterfoht, Bernd Stannowski, Heinz Christoph Neitzert, René A. J. Janssen, Christian Michael Wolff, Dieter Neher, Felix Lang**

Guorui He, Andrés-Felipe Castro-Méndez, Paria Forozi Sowmeeh, Dieter Neher, Felix Lang
Soft Matter Physics and Optoelectronics, Institute of Physics and Astronomy, University of Potsdam, Karl-Liebknecht-Str. 24–25, 14476 Potsdam-Golm, Germany
E-mail: felix.lang.1@uni-potsdam.de (Felix Lang); andres.castro@uni-potsdam.de (Andrés-Felipe Castro-Méndez)

Jonas Diekmann, Christian Michael Wolff
École Polytechnique Fédérale de Lausanne (EPFL), Institute of Electrical and Microengineering (IEM), Photovoltaics and Thin Film Electronics Laboratory (PV-Lab), Rue de la Maladière 71b, 2000 Neuchâtel, Switzerland

Guus J. W. Aalbers, Simon V. Quiroz Monnens, René A. J. Janssen
Molecular Materials and Nanosystems & Institute for Complex Molecular Systems, Eindhoven University of Technology, P.O. Box 513, 5600 MB Eindhoven, The Netherlands

René A. J. Janssen
Dutch Institute for Fundamental Energy Research, De Zaale 20, 5612 AJ Eindhoven, The Netherlands

Arpana Singh, Heinz Christoph Neitzert
Department of Industrial Engineering (DIIN), University of Salerno, 84084 Fisciano, Italy

Francisco Peña-Camargo

Helmholtz-Zentrum Berlin für Materialien und Energie, Solar Energy Division, 12489 Berlin, Germany

Martin Stolterfoht

Electronic Engineering Department, The Chinese University of Hong Kong, Sha Tin N.T., Hong Kong SAR, China

Bernd Stannowski

PVcomB, Helmholtz-Zentrum Berlin, 12489 Berlin, Germany

Beuth University of Applied Sciences Berlin, 13353 Berlin, Germany

Experimental Section

Device Fabrication

Perovskite solar cells were prepared with the layer sequence glass/ITO/CbzNaph/$Cs_{0.21}FA_{0.74}MA_{0.05}PbI_{0.81}Br_{0.14}Cl_{0.05}$/PIC/$C_{60}$/BCP/Cu, where the CbzNaph and BCP represent (4-(7H-dibenzo[c,g]carbazol-7-yl)butyl)phosphonic acid and bathocuproine, respectively. First, the patterned ITO-coated glass was ultrasonically cleaned via Hellmanex (30 min), distilled water (20 min), acetone (7min), and isopropanol (10 min), and then treated in ultraviolet ozone for 30 min before being placed in a glove box with nitrogen atmosphere. Then, the CbzNaph with a concentration of 0.5 mg/mL in ethanol was first sonicated for 10 min and then deposited with a spinning speed of 3000 rpm for 30 s, followed by annealing at 100 °C for 10 min. After cooling down to room temperature, the perovskite solution with a concentration of 1.4 M in a mixed solvent of DMF and DMSO (DMF:DMSO=3:1 in volume ratio) was deposited by spin coating at 5000 rpm for 50 s (2 s for acceleration). 25 s before the end of spin coating, 250 μL chlorobenzene as antisolvent was dispensed onto the perovskite layer, followed by annealing at 100 °C for 30 min. The polymer point contacts were formed by depositing 80 μL blend solution of polystyrene (PS) from Sigma-Aldrich with a molecular mass of 35 kDa and polymethyl methacrylate (PMMA) from Sigma-Aldrich with M_w of 15000 with a concentration of 25mg/mL with different weight ratios of PS:PMMA=1:0.5, 1:1, 1:2, 1:3 and 1:5 in chlorobenzene on the already cool-down perovskite layer at 5000 rpm for 40 s. Afterwards, 100 μL ortho-xylene as a selectively PS-dissolving solvent was deposited to remove the PS with the same spin-coating condition as that of polymer solution. For the thin PS or PMMA layer passivated devices, the beforementioned PS or PMMA was dissolved in chlorobenzene with a concentration of 1 mg/mL and was deposited on top of the perovskite layer with the same spin coating conditions. For the "PEAI before" or "PEAI after" device, phenethylammonium iodide (PEAI) from Sigma-Aldrich was dissolved in isopropanol with a concentration of 1 mg/mL and was deposited before or after the point contacts were formed with the same spin-coating condition as that of polymer solution. For the "LiF before" or "LiF after" device, 0.8 nm LiF was thermal-evaporated with a rate of 0.05 Å/s before or after the point contacts were formed. Then, the samples were transferred to an evaporation chamber, where 30 nm C_{60} at 0.2 Å/s, 8 nm BCP at 0.2 Å/s and 100 nm copper at 0.1-1 Å/s, were deposited under vacuum to complete the device with an active area of 0.0648

cm^2, which is defined by the metal shadow mask and the patterned ITO. For another recipe with the device structure of glass/ITO/CbzNaph/$Cs_{0.05}FA_{0.98}MA_{0.02}Pb(I_{0.98}Br_{0.02})_3$/patterned LiF/$C_{60}$/BCP/Cu, the perovskite solution in a mixed solvent of DMF and DMSO (DMF:DMSO=5:1 in volume ratio) was deposited by spin coating at 1000 rpm for 10 s followed by 5000 rpm for 40s. 12 s before the end of spin coating, 250 µL of chlorobenzene as antisolvent was dispensed onto the perovskite layer, followed by annealing at 100 °C for 20 min. The patterned LiF was formed by attaching a fine Nylon 180 mesh (SPI Supplies) to the surface of the perovskite and then evaporating 5 nm LiF. The other processing methods are the same as the $Cs_{0.21}FA_{0.74}MA_{0.05}PbI_{0.81}Br_{0.14}Cl_{0.05}$ recipe.

Characterization

J-V characteristics measurement:

The *J-V* characteristics of the devices were measured with a Keithley 2400 system and a filtered Oriel Class AAA Xenon lamp, which is calibrated to 100 mW cm^{-2}, was used to simulate the sun illumination. The temperature of the devices was controlled at 25 °C with a sample holder with temperature controller.

External quantum efficiency (EQE_{PV}):

A Philips halogen lamp with a power of 300 W was used as light source and the light was chopped by 80 Hz with a chopper and gets mono-chromated with an Oriel Cornerstone 260 monochromator. The EQE_{PV} spectra were calibrated by a silicon diode (Newport UV 818) until 1000 nm. The signal was sent to the pre-amplifier (Stanford Research SR570) and read out by an analogue lock-in amplifier (Stanford Research SR830).

Sensitive external quantum efficiency (EQE_{PV}):

For regular EQE_{PV} measurements, a tungsten halogen lamp (Philips Focusline, 50 W) was used as a light source and its light was chopped at 165 Hz with a mechanical chopper (Stanford Research SR540) before passing through a monochromator (Oriel Cornerstone 130) and an aperture (0.0314 cm^2). The response of the solar cell was measured using a low-noise current pre-amplifier (Stanford Research SR570) in combination with a lock-in amplifier (Stanford Research SR830). The incident light intensity was referenced with a Si detector. Sensitive EQE_{PV} measurements were conducted with the light from an Osram 64655 HLX 250 W

tungsten halogen lamp. The light was mechanically chopped at 333 Hz and passed through appropriate sorting filters and dispersed with an Oriel Cornerstone 260 monochromator. The response was amplified using a Stanford Research SR570 pre-amplifier and a Stanford Research SR830 lock-in amplifier. Calibration was performed using reference Si at shorter wavelengths and InGaAs detectors at longer wavelengths. The measured highly sensitive EQE_{PV} spectra were stitched to regular EQE_{PV} data.

Atomic force microscopy (AFM) and conductive-AFM (c-AFM):

A halogen lamp was used as a light source and an incorporated microscope was used to observe the probing area. The morphology and height were measured using a standard scanning force microscopy (SFM) system (MultiView 1000, Nanonics Imaging). For the c-AFM measurements, platinum/iridium5 coated tip (CONTPt-20 from NanoWorld) was utilized. A voltage of -10 V was applied from the tip, and the current was read out via the system.

Photoluminescence (PL) and photoluminescence quantum yield (PLQY):

For the PL measurements, the samples were encapsulated by thin transparent films along the edges. An Andor Solis 803 System composed of mirrors and filters was used. A silicon detector with a 670 nm center wavelength (CWL) grid were equipped to detect the PL intensity. An excitation wavelength of 520 nm laser was used and the light was adjusted to one-sun equivalent intensity according to the specific bandgap of the absorber and the intensity was kept the same for all the samples. The PLQY measurements were performed inside an integrating sphere (Hamamatsu Photonics K.K. A10094) under the illumination of a 520 nm continuous laser (InsaneWare). The intensity of the laser was adjusted to one-sun equivalent intensity using a Si photodiode. PLQY can be calculated as the ratio of the number of photons emitted to the number of photons absorbed by the samples. The emitted and absorbed photons can be determined by performing different measurements:

1. Without sample in the integrating sphere, with laser filter for remaining laser correction.
2. With sample in the integrating sphere, without laser filter for absorptance.
3. Without sample in the integrating sphere, without laser filter for reference.
4. With sample in the integrating sphere, with laser filter for emission.

Electroluminescence (EL) and electroluminescence quantum yield (ELQY):

For EL measurements, a constant voltage was applied to the device using a Keithley 2400 to reach the same current density as the short-circuit current density under AM 1.5G, 100 mW cm^{-2}. Emission spectra were recorded by an Andor Solis 803 System with a silicon detector with a CWL of 670 nm. For the ELQY measurements, a Hamamatsu silicon photodiode 1010B was placed in front of the measured pixel with a distance smaller than 0.5 cm. A Keithley 485 Picoammeter was connected to the detector and used to measure the emitted light intensity. A Keithley 2400 SourceMeter was used to apply the voltage and record the injected current. ELQY can be calculated as the ratio of J_{detector} to $J_{\mathrm{injection}}$, where J_{detector} is the photocurrent of the detector, $J_{\mathrm{injection}}$ is the injected current of the device. Then, the ELQY value is multiplied by a conversion factor (1.31 in this work, see Fig. S1), considering the emission spectrum of the device and the external quantum efficiency of the detector.

Photoluminescence (PL), electroluminescence (EL) imaging, reflection imaging:

For photoluminescence (PL) imaging, a fluorescence microscope (Olympus BX51) was utilized and a built-in halogen lamp powered by Olympus U-RFL-T-200 was used as the light source. The samples were held in a home-built holder and illuminated with a green light with a wavelength of 510-550 nm. A CCD camera (Hamamatsu Photonics C4742-80-12AG) with a long-pass filter (> 590 nm) was used to image the luminescence from the perovskite device. The exposure time was adjusted to obtain the optimal signals. For electroluminescence (EL) imaging, the light source was blocked and a Keithley 2400 system was used to apply the voltage and record the injected current. The injected current is equal to the short-circuit current of the devices under AM 1.5G, 100 mW cm^{-2} illumination. For reflection imaging, the samples were illuminated with white light and the reflected light was imaged without using any filter.

Resistance-dependent photovoltage (RPV):

The laser pulse comes from a diode pumped, Q-switched Nd:YAG laser (NT242, EKSPLA) with a ~5 ns pulse duration at a frequency of 5 Hz. Photovoltage transients were recorded by an oscilloscope (Agilent DSO9104H) with a load resistance of 100M ohm. Low fluences of the laser pulse were used to avoid screening of the internal field and build-up of charges inside the device.

Time-resolved microwave conductivity (TRMC):

A TRMC setup, which operates in the Ka-band, consisting of a Gunn-diode source, a circulator and a fast diode as receiver, was used. The sample was placed on the open waveguide and exposed to a short laser pulse with a wavelength of 520 nm (Thorlabs NPL52C) with a pulse length of 129 ns. The photo-generated charge carriers cause a change in the sample conductivity. Consequently, the reflected microwave power changes accordingly. The reflected microwave power is directed via the circulator to the fast detector. After appropriate amplification, the resulting transient signal is recorded via a fast digitizer.

Dark-charge extraction by linearly increasing voltage (dark-CELIV):
In dark-CELIV the device was initially held at short-circuit condition. Then the voltage was linearly increased from 0 V to -0.4 V (in reverse bias) using a pulse generator. The slope (A) was varied to assess a wide timescale range. The current transients were recorded with an oscilloscope (Agilent DSO9104H) and measured with a variable load resistance (R), which was adjusted as 20 Ω at short 10 μs pulse and up to 1 MΩ at 0.3 s pulses to keep the voltage response approximately constant. The increased load resistance loses the time resolution at short times but allows to record the response for long time range. The continuous accumulation of the charges at the electrodes leads to a step-like voltage response. The voltage response step of the solar cell is calculated as $\Delta V = A \times R \times C$, from which we calculated the capacitance of the cell (C). Mobile ions in the active layer can lead to an additional bump in the voltage response.[1]

Fast hysteresis (FH) measurement:
Fast J-V curves were obtained by applying a triangular voltage pulse to the solar cells starting from approximately V_{OC} to 0 V and back to V_{OC}. The scan speed time was varied and the voltage response was measured with an Agilent DSO9054H High-Definition Oscilloscope, using an external load resistance of 3 Ω. The voltage pulse was supplied by an Agilent 33220A function generator in combination with a home-built amplifier (4x).

X-ray diffraction (XRD) measurement:
X-ray diffraction (XRD) spectra were obtained by using Bragg Brentano geometry using a Panalytical Empyrean.

Stability test:

For stabilized maximum power output (SPO) stability test, the encapsulated devices were kept at maximum output voltage at 35 °C in air under one-sun equivalent illumination and a *J-V* curve was measured with a reverse scan at a certain time interval. For the maximum power output (MPP) test, the devices were kept at maximum power output at 25 °C under nitrogen inside a glovebox under one-sun equivalent intensity. For both stability tests, Cree CXB3590 lamps (LiEnTec GmbH) was used.

Supplementary Note

Supplementary Note 1. Quantification of the QFLS[2]

The Shockley-Queisser equation links the radiative recombination current density of free charges (J_{rad}) to the chemical potential per free electron-hole pair (μ) or the quasi-Fermi level splitting (QFLS) in the active material[3, 4] as shown as follows:

$$J_{rad} = J_{0,\ rad} \exp (\mu/k_B T) \quad (1)$$

Here, $J_{0,\ rad}$ is the radiative thermal recombination current density in the dark condition, k_B is the Boltzmann constant, T is the temperature. If radiative recombination originates only from free charges, then the radiative recombination current equals the photoluminescence yield per second times the elementary charge. Photoluminescence quantum yield (PLQY) can be calculated as the ratio of radiative recombination to total recombination ($J_{R,\ tot}$). Under open-circuit condition, total recombination is equal to total generation (J_G), and therefore,

$$\mathrm{PLQY} = J_{rad}/J_G \quad (2)$$

Now, by using the J_{rad} in Equation 1 to substitute the one in Equation 2, we can relate the QFLS to the measured PLQY as

$$\mu = k_B T \ln (\mathrm{PLQY} \times J_G/J_{0,\ rad}) \quad (3)$$

Electroluminescence quantum yield (ELQY) can be calculated as the ratio of radiative recombination to total injection ($J_{injection}$). When the injection is equal to the total generation at one-sun intensity, the same equation as PLQY can be used, so

$$\mathrm{ELQY} = J_{rad}/J_G \quad (4)$$

Similarly, by substituting the J_{rad}, we can relate QFLS_{EL} to the measured ELQY as

$$\mathrm{QFLS}_{EL} = k_B T \ln (\mathrm{ELQY} \times J_G/J_{0,\ rad}) \quad (5)$$

We used the corresponding J_{SC} of the device as J_G. The calculation of $J_{0,\,rad}$ is shown in Fig. S8.

Point Contact Simulation

Simulation S1. FH measurements via 1-D drift-diffusion simulation

A one-dimensional model of a perovskite solar cell was simulated using SETFOS, where the drift-diffusion equations were solved for each layer based on predefined material and device parameters. The simulation incorporated mobile ion migration, assuming the presence of only a single mobile ion species, which was confined exclusively to the perovskite layer. To analyze FH (fast hysteresis) behavior, a transient measurement was performed using a voltage ramp, sweeping from 0V to open-circuit voltage and back to 0V at multiple scan rates.

Simulation S2. Mobile ions distribution via 2-D drift-diffusion simulation

To simulate the electronic behavior of perovskite solar cells in two dimensions, the semiconductor module of Comsol Multiphysics was used. As for 1-D simulations, the optoelectronic parameters of each layer are defined, which allows for solving the Drift-Diffusion equation for electrons and holes. To facilitate the simulation of mobile ions, the Semiconductor module is coupled to a custom partial differential equation module that contains the Drift-Diffusion equation of mobile ions.[5] Mobile ions were assumed to migrate only within the perovskite itself and limited to one mobile ion species. Partial contacts were realized by assuming insulating boundary conditions at the side and bottom of the valleys.

Supplementary Figure

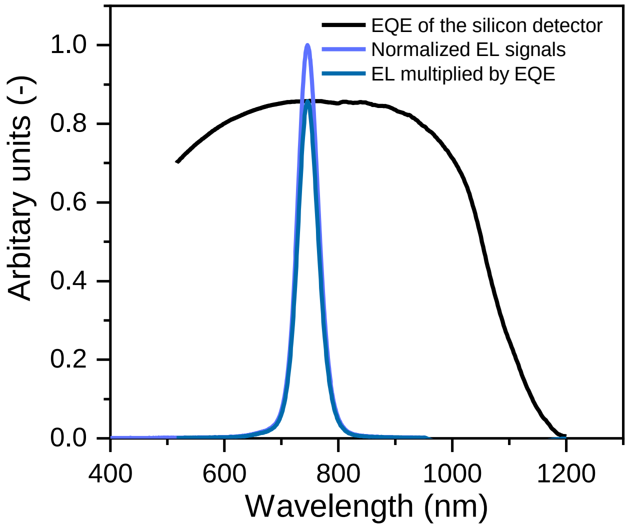

Fig. S1. The EQE_{PV} spectra of the silicon detector, the normalized EL spectra of the "control" device and the product of the EL and the EQE_{PV} spectra. Dividing the area of the normalized EL by that of the product of EL and the EQE_{PV} spectra gives the conversion factor of 1.31.

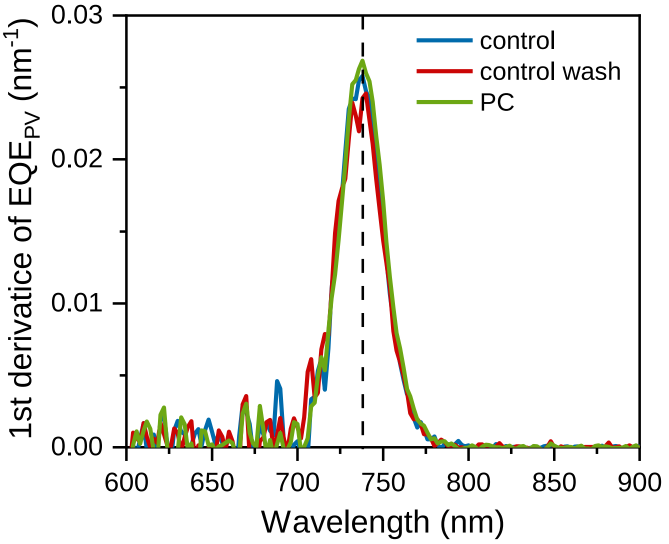

Fig. S2. The first derivative of EQE_{PV} (Fig. S10) with respect to the wavelength from which the photovoltaic bandgap of the device is determined as 1.68 eV.

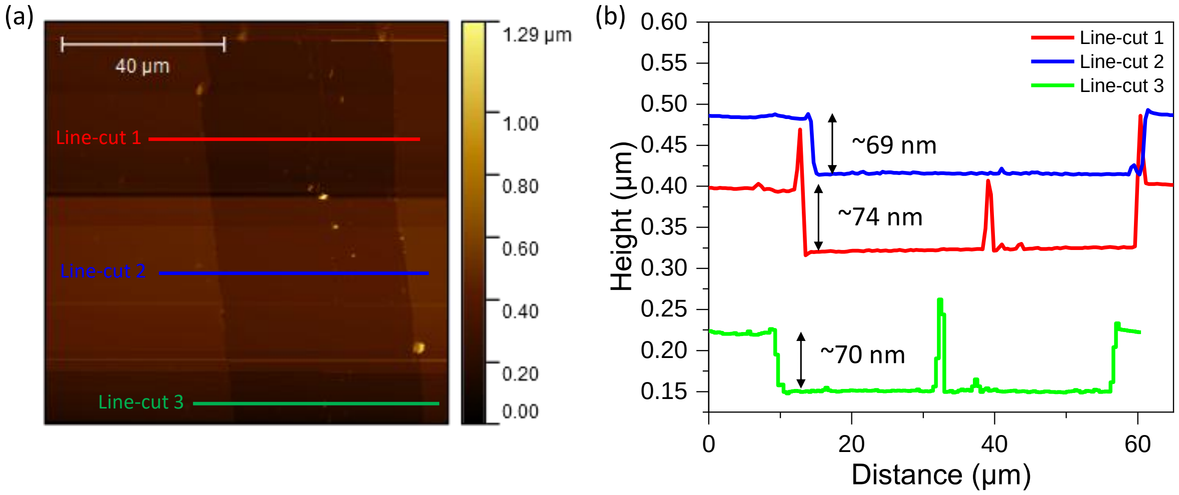

Fig. S3. Atomic force microscopy (AFM) images of a compact polymer layer on top of a silicon substrate. The polymer layer was formed by depositing a blend solution of PS and PMMA with a concentration of 25 mg/mL without o-xylene washing. A scratch was made across the film for the thickness measurement. The thickness of the film was determined as around 70 nm according to the results of three different line cuts. We note that the thickness of PMMA on different surfaces, perovskite or silicon surface, can vary.

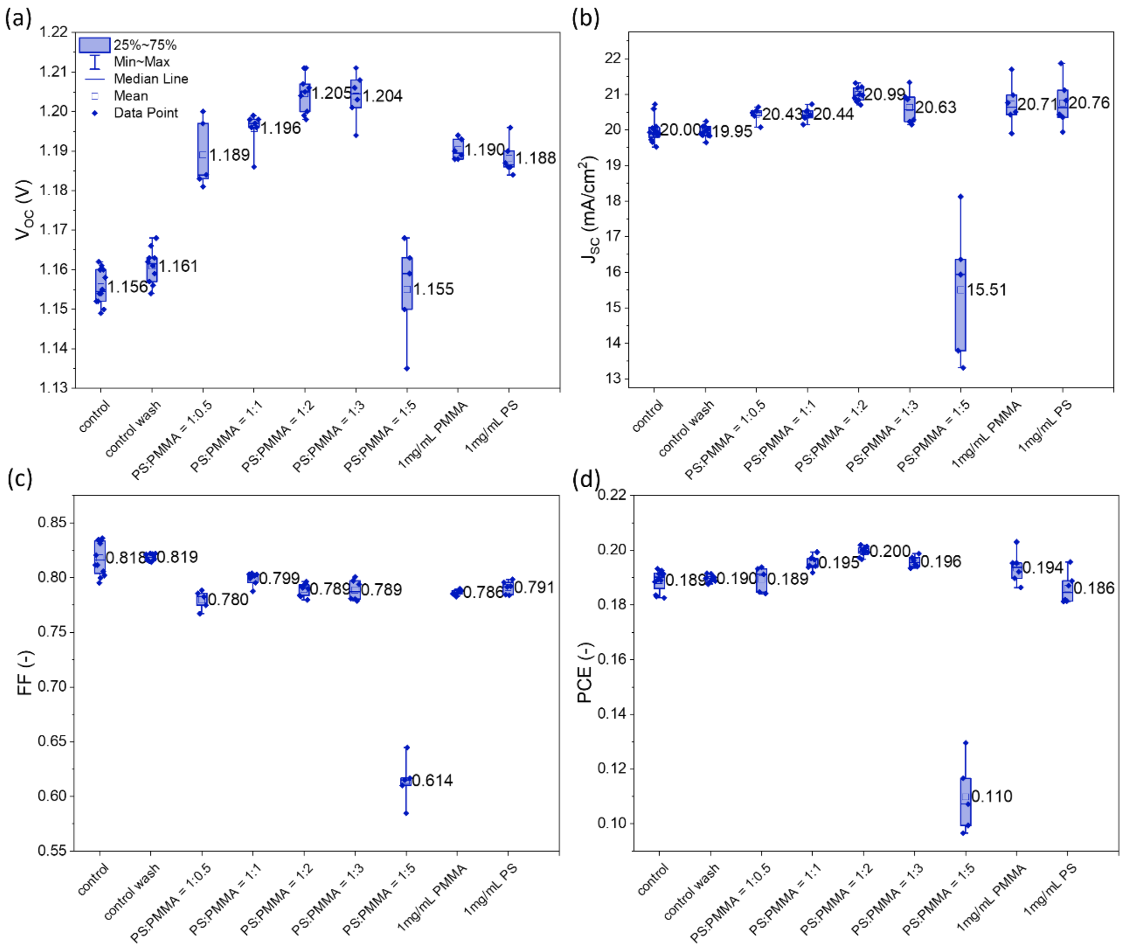

Fig. S4. The box diagram of (a) V_{OC}, (b) J_{SC}, (c) FF and (d) PCE of "control", "control wash", PC PSCs with different weight ratios of PS and PMMA and thin polymer layer passivated devices under AM 1.5G, 100 mW cm^{-2} illumination with scatters of data points.

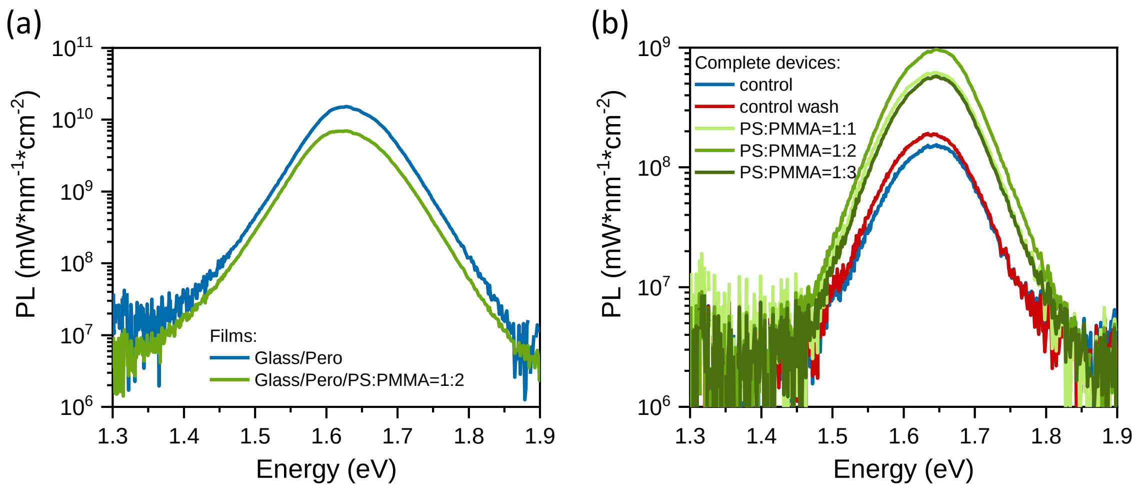

Fig. S5. The photoluminescence spectra of the (a) films on glass substrates and (b) complete device stacks under one sun intensity with a 520 nm laser.

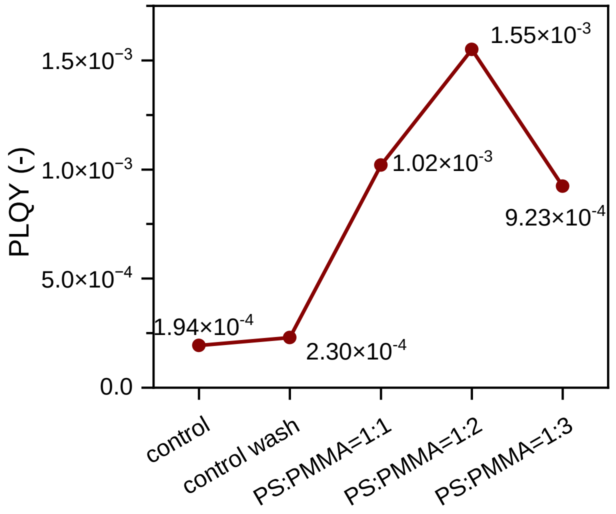

Fig. S6. The PLQY values for devices with different conditions. The PLQY values of the "control" and "control wash" devices are comparable, and increase when a weight ratio of PS and PMMA is reduced from 1:1 to 1:2.

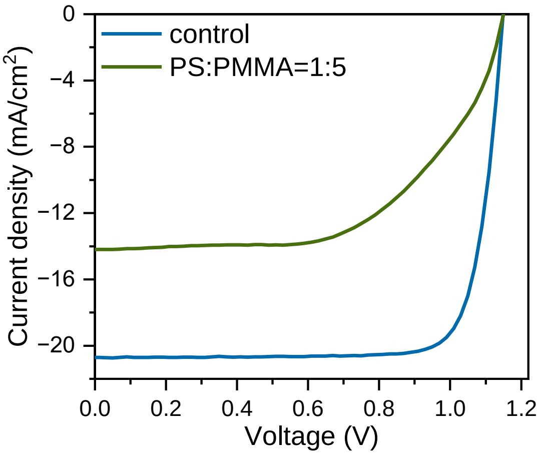

Fig. S7. Current density-voltage (*J*-*V*) curves of the "control" device and PS:PMMA=1:5 treated device under illumination (AM 1.5G, 100 mW cm^{-2}) measured with a reverse scan. Compared with "control" device, PS:PMMA=1:5 treated device exhibits much lower J_{SC} and FF, indicating extraction problems.

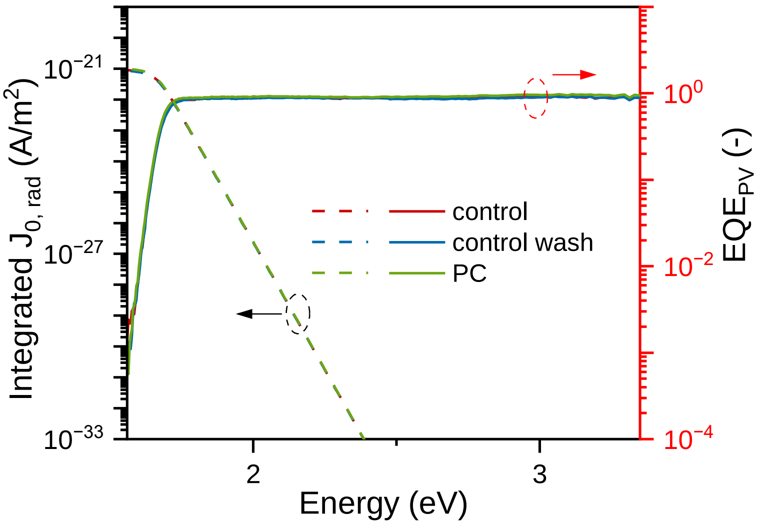

Fig. S8. The $J_{0,\,rad}$ of the "control", "control wash" and PC device is calculated as 9.06×10^{-22} A/m^2, 8.48×10^{-22} A/m^2 and 9.75×10^{-22} A/m^2, respectively, by integrating the EQE times the black body emission over energy.

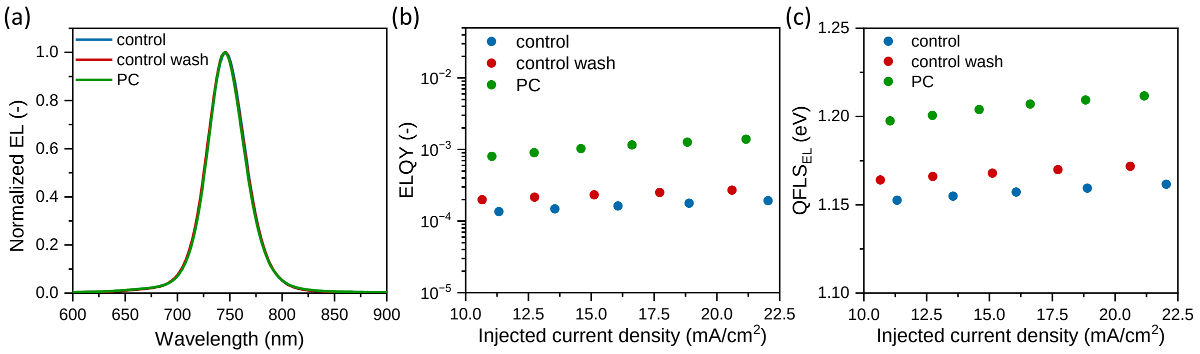

Fig. S9. (a) The electroluminescence spectra, (b) the electroluminescence quantum yield (ELQY) values of 1.93 × 10^{-4}, 2.69 × 10^{-4} and 1.38 × 10^{-3}, (c) the extracted $QFLS_{EL}$ values of 1.16 eV, 1.17 eV and 1.21 eV for the "control", "control wash" and PC device, respectively. The ELQY values are obtained at the injected current density equal to the J_{SC} of the devices. Comparable results are obtained for the "control" and "control wash" devices.

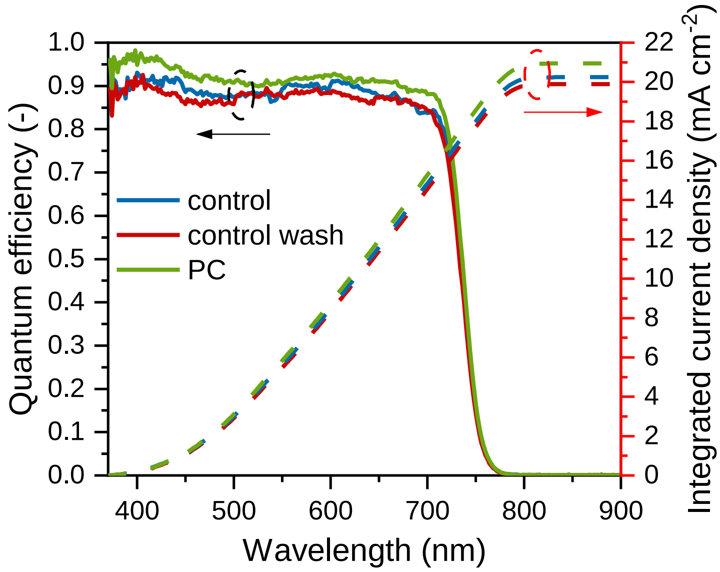

Fig. S10. EQE_{PV} spectra of the "control", "control wash" and PC device with the corresponding current density curve integrated from the EQE_{PV} data. The J_{SC} calculated from the integration of EQE_{PV} multiplied by solar spectrum matches well with the J_{SC} measured under AM 1.5G, 100 mW cm^{-2} illumination, with an error within ± 3%.

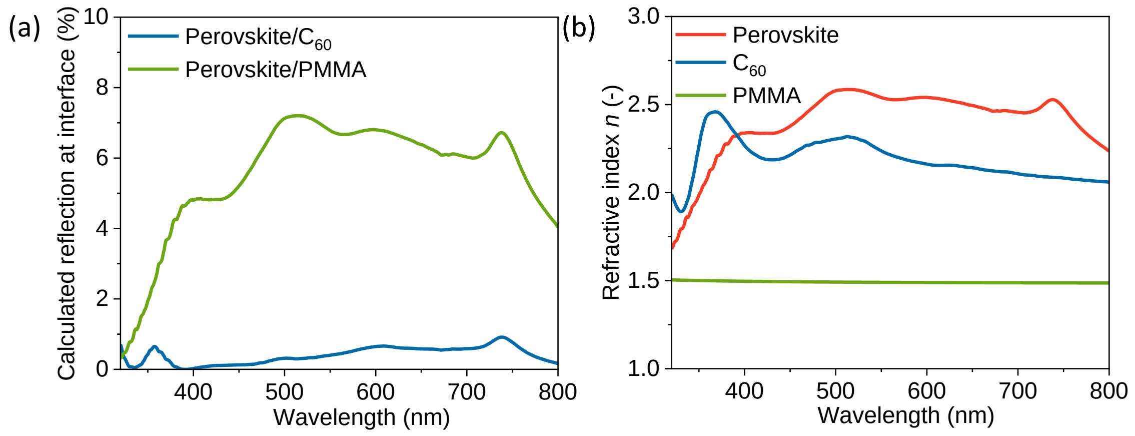

Fig. S11. (a) The calculated reflection at perovskite/C_{60} and perovskite/PMMA interface. (b) The refractive index of perovskite, C_{60} and PMMA, which are used for the reflection calculated in panel (a) according to Fresnel reflection equation.

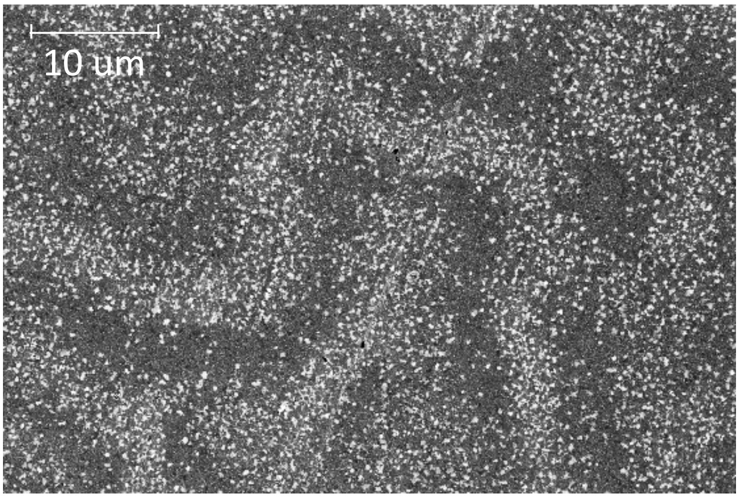

(a) control

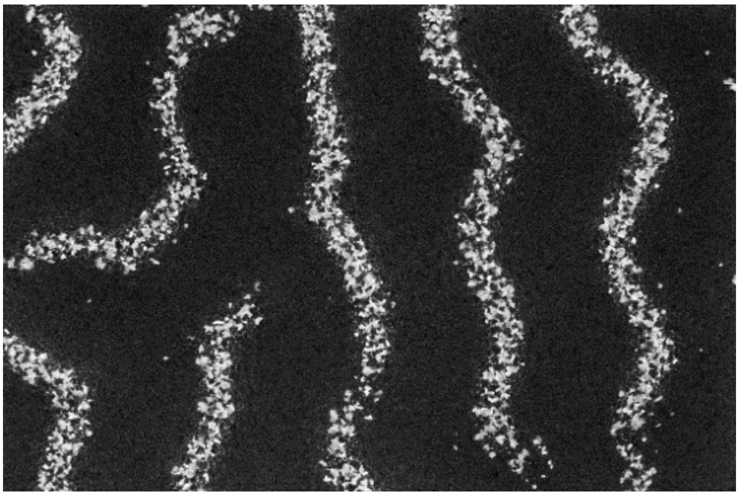

(b) control wash

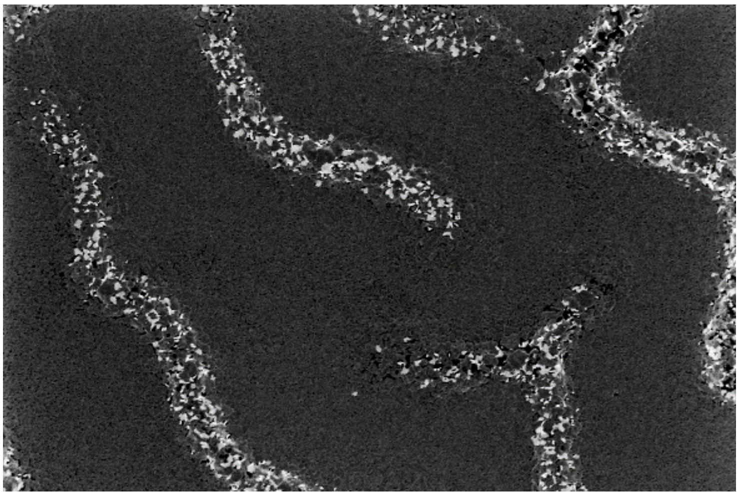

(c) PS:PMMA=1:2

Fig. S12. Top-view scanning electron microscopy (SEM) images of (a) “control”, (b) “control wash” and (c) PC (PS:PMMA=1:2) perovskite film on a fully-covered ITO substrate with the

immersion lens (in-lens) detector. The brighter crystals are lead iodides due to its higher average atomic number.[6] We determine the brighter regions, where more lead iodide accumulates, as the mountain-like regions by measuring SEM and AFM images of the “control wash” film in the same area, as shown in Fig. S13.

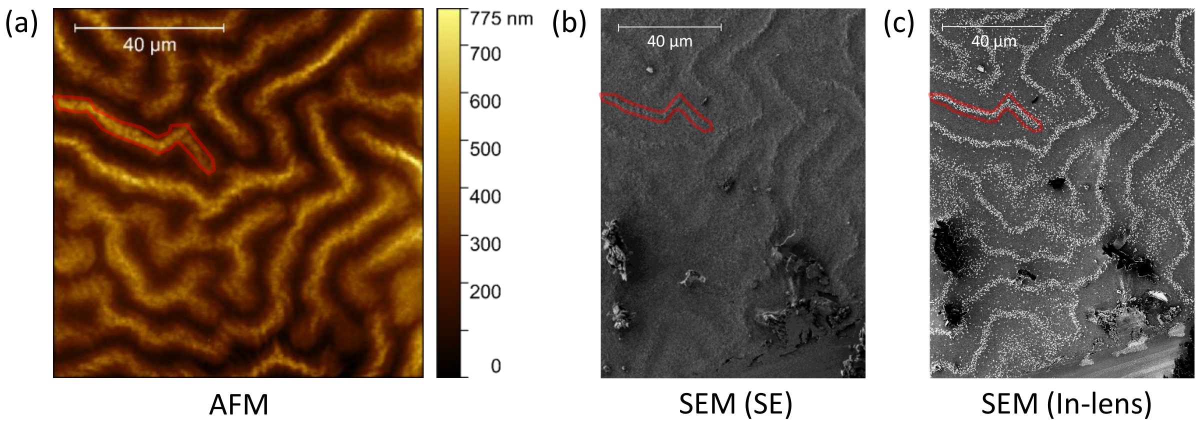

Fig. S13. (a) Atomic force microscopy (AFM) image, (b) SEM image with the secondary electron (SE) detector and (c) SEM image with the in-lens detector of the “control wash” perovskite film on a fully covered ITO substrate at the same area. One identical “mountain” feature is marked with a red contour.

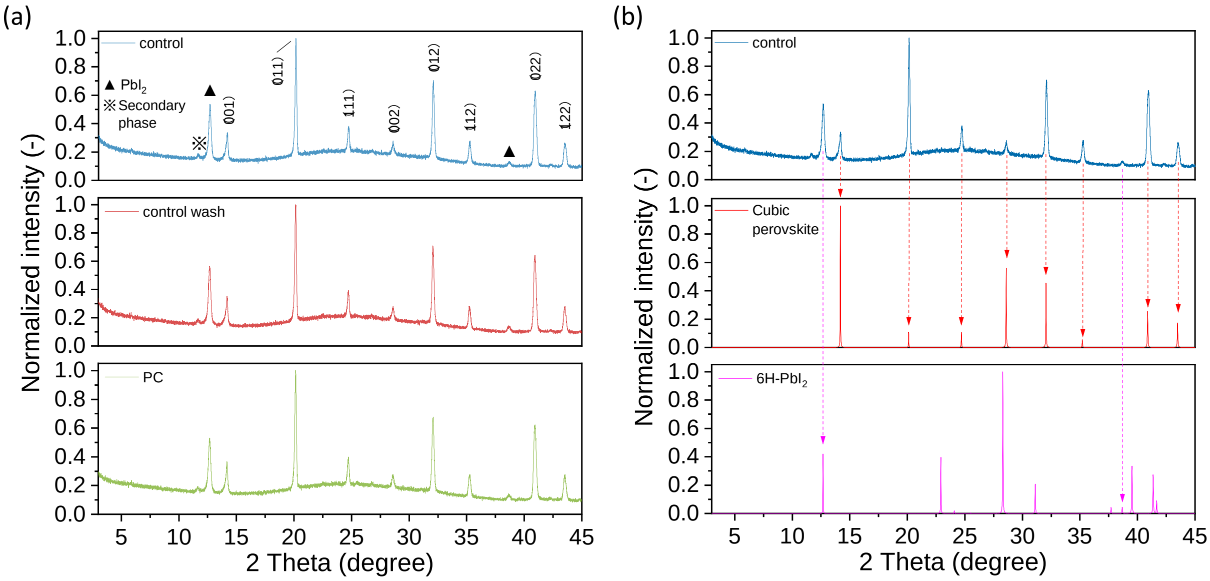

Fig. S14. (a) X-ray diffraction (XRD) spectra of the “control”, “control wash” and PC perovskite film on a glass substrate. (b) XRD spectra of the “control” film in comparison to simulated XRD spectra of cubic halide perovskite with a lattice parameter a = 6.2386 Å and 6H-PbI_2 phase for diffraction patterns determination.

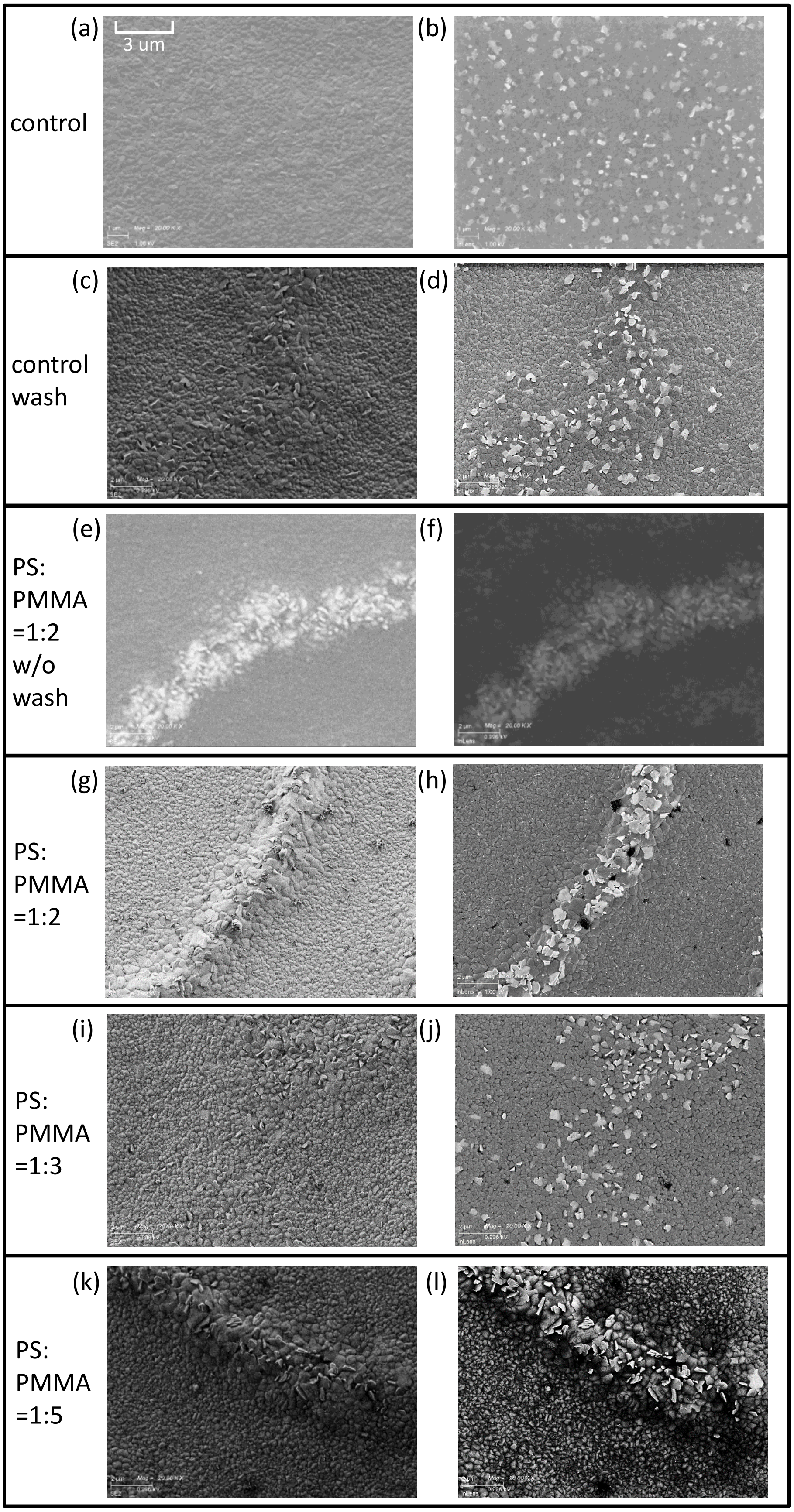

Fig. S15. Top-view scanning electron microscopy (SEM) images with secondary electron (SE) detector of (a) “control”, (c) “control wash”, (e) PS:PMMA=1:2 on perovskite film without

ortho-xylene treatment, (g) PC (PS:PMMA=1:2), (i) PS:PMMA=1:3, (k) PS:PMMA=1:5 on a fully-covered ITO substrate and SEM images with immersion lens (in-lens) detector of (b) “control”, (d) “control wash”, (f) PS:PMMA=1:2 on perovskite film without ortho-xylene treatment, (h) PC (PS:PMMA=1:2), (j) PS:PMMA=1:3, (l) PS:PMMA=1:5. SE detector is more surface-sensitive than in-lens detector. More blurry images are observed in (e) and (f), when the polymer (PS:PMMA=1:2) is deposited on the perovskite surface without ortho-xylene washing, since a thick polymer layer can scatter the incident electrons and reduce the resolution. After the ortho-xylene washing, PMMA clusters are observed in (g), (i) and (k), and shown as dark features in (h), (j), (l) while such features are not observed in (a), (b) “control” or (c), (d) “control wash” film.

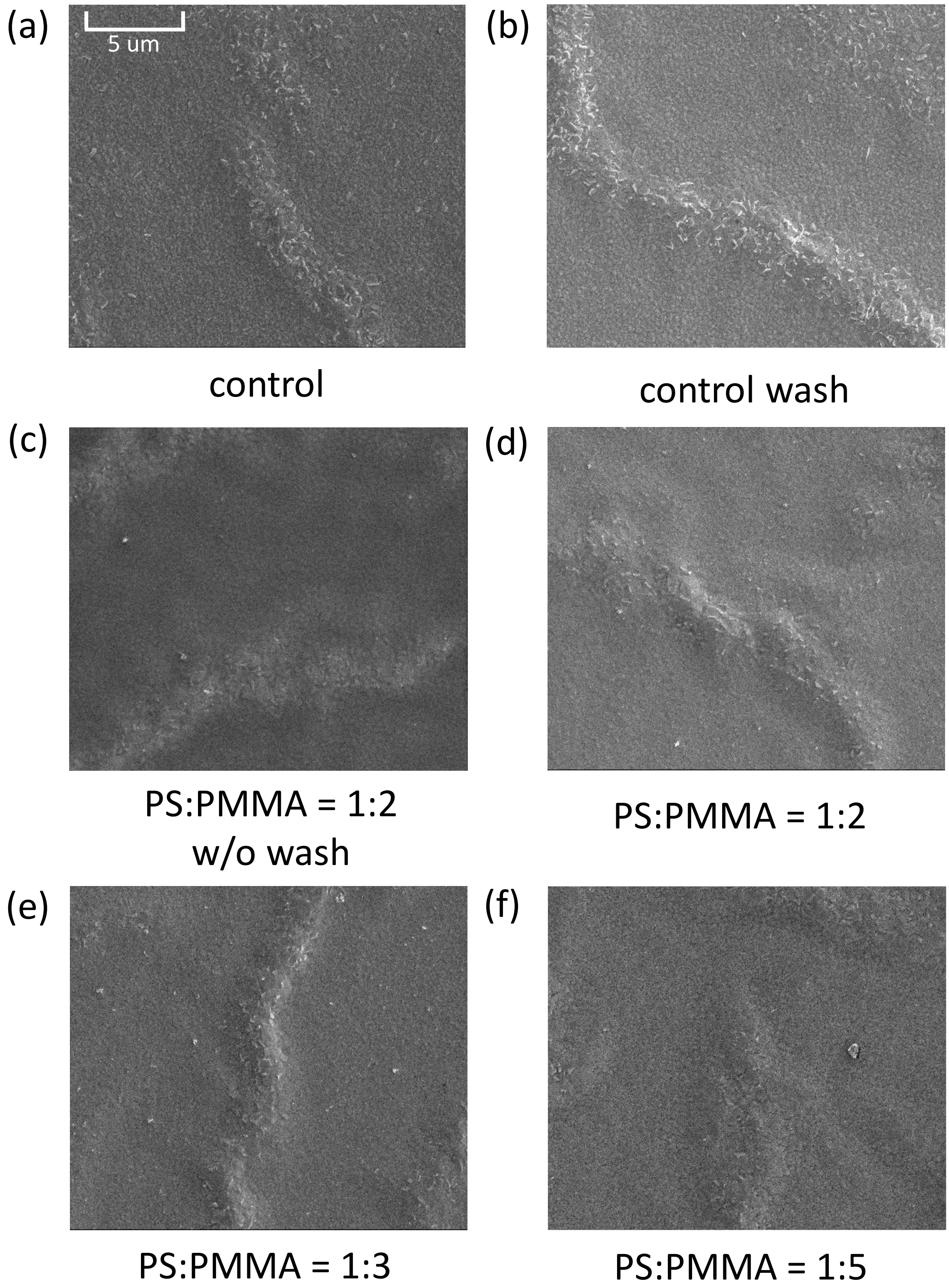

Fig. S16. Top-view scanning electron microscopy (SEM) images with secondary electron (SE) detector of (a) “control”, (b) “control wash”, (c) PS:PMMA=1:2 on perovskite film without ortho-xylene treatment, (d) PC (PS:PMMA=1:2), (e) PS:PMMA=1:3, (f) PS:PMMA=1:5 on a

fully-covered ITO substrate. Note that a thin layer of gold is sputtered on top of these films to resolve the topography. Clearer perovskite grains are observed in (a) and (b). When the polymer (PS:PMMA=1:2) is deposited on the perovskite surface without ortho-xylene washing, overall resolution on the perovskite grains is reduced, as shown in (c). After the ortho-xylene washing, PMMA remains and covers partially the perovskite and a higher coverage is observed with an increasing weight ratio of PMMA, as shown in (d), (e) and (f).

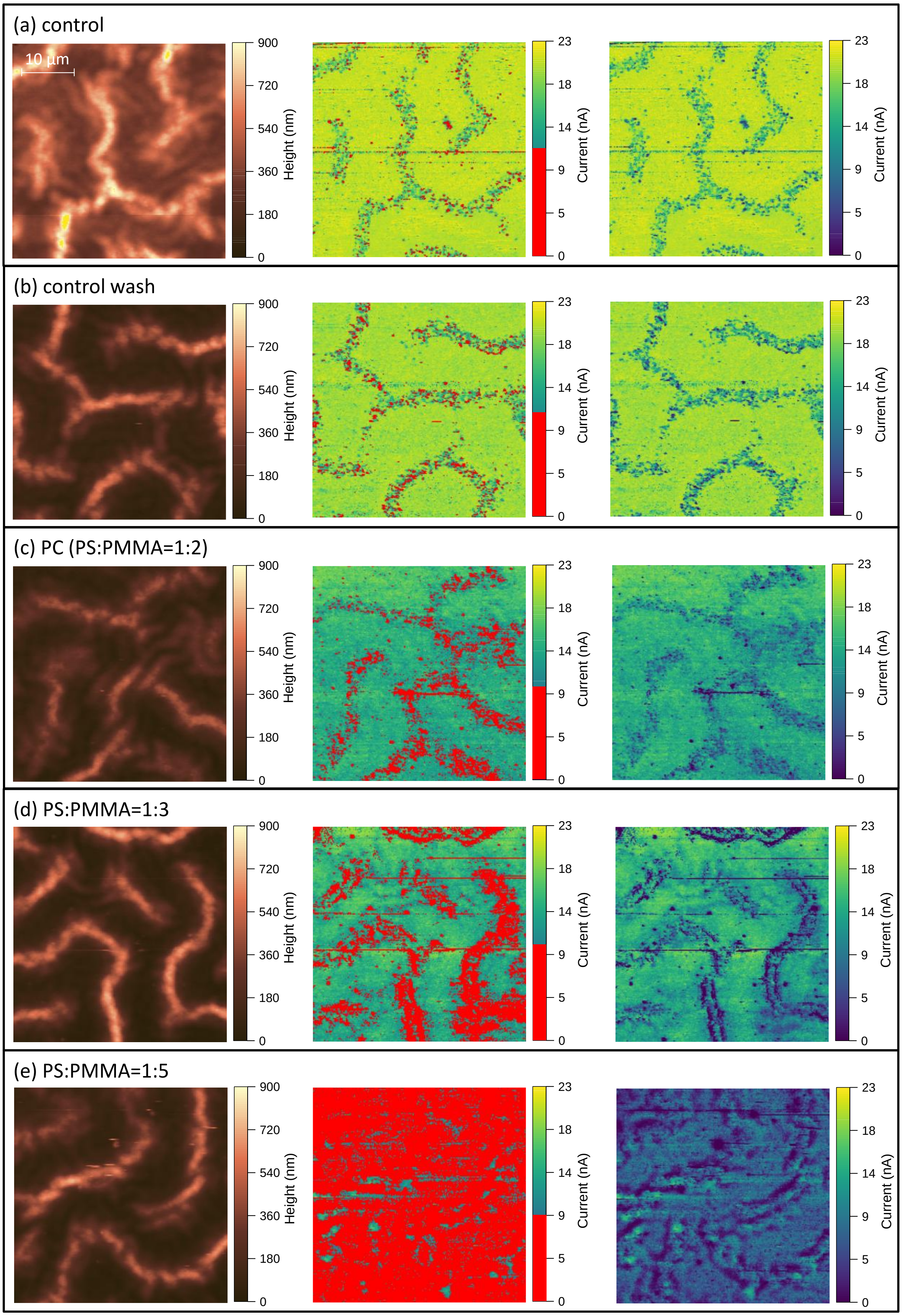

(a) control
10 μm
900
720
540
360
180
0
Height (nm)
23
18
14
9
5
0
Current (nA)
(b) control wash
(c) PC (PS:PMMA=1:2)
(d) PS:PMMA=1:3
(e) PS:PMMA=1:5

Fig. S17. Atomic force microscopy (AFM) at left, conductive atomic force microscopy (c-AFM) images with current below half of its maximum current marked as red at middle and the raw data of c-AFM at right of the (a) “control”, (b) “control wash”, (c) PS:PMMA=1:2, (d) PS:PMMA=1:3, (e) PS:PMMA=1:5 based perovskite film with C_{60} on top on a fully covered ITO substrate.

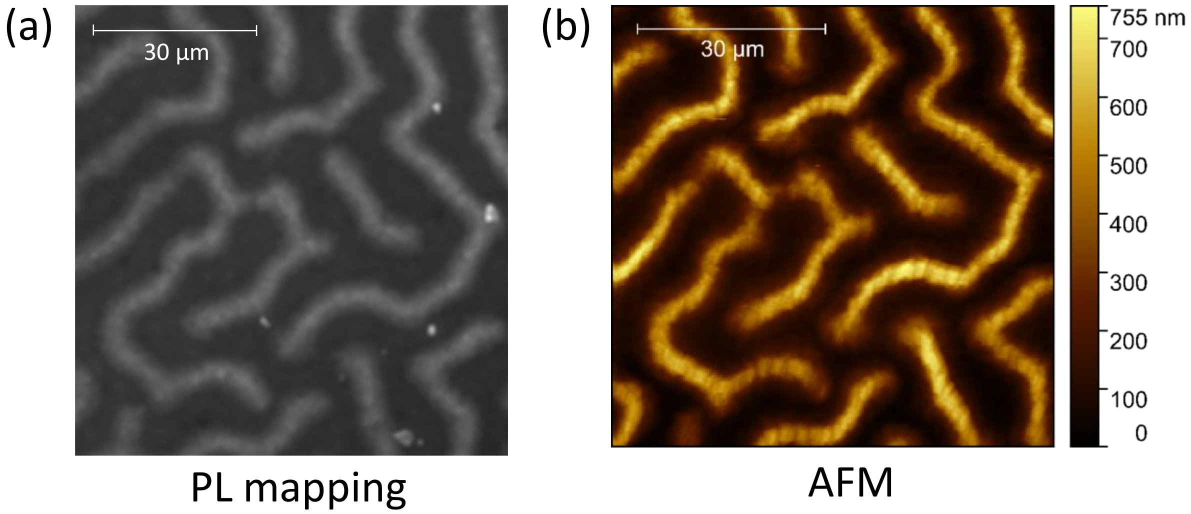

Fig. S18. (a) The photoluminescence (PL) image and (b) AFM image of the "control wash" perovskite film at the same spot. All images here were taken from the perovskite side. By comparing the identical features in the PL and AFM images, we find out that the more emissive regions in the PL images correspond to the "mountain" regions.

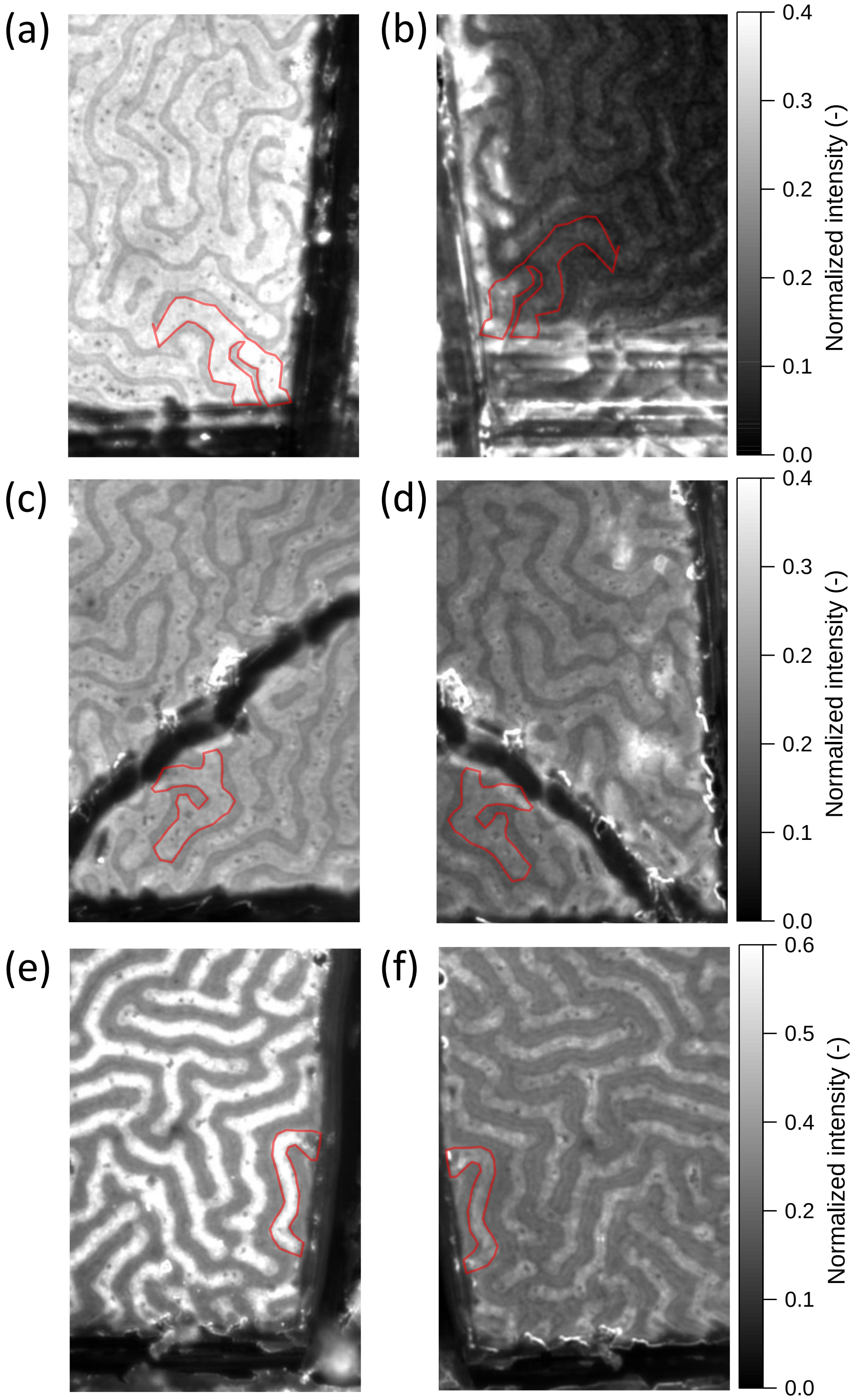

Fig. S19. PL images of "control" film with incident light (a) from the perovskite side and (b) from the glass side, "control wash" film (c) from the perovskite side and (d) from the glass side, PC film (e) from the perovskite side and (f) from the glass side. The identical features are marked with red contours. The relative PL intensity is not dependent on the illumination incident direction.

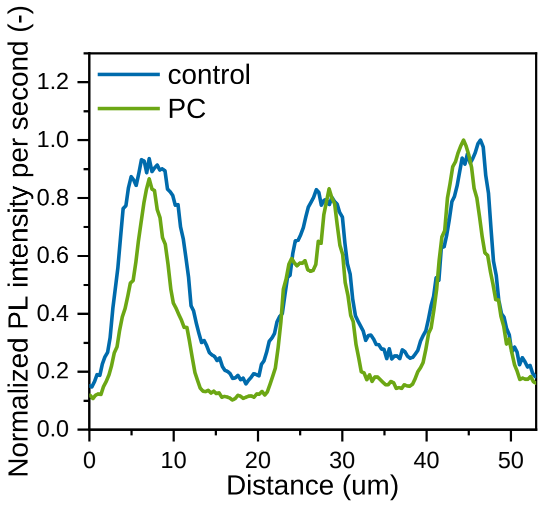

Fig. S20. Normalized PL intensity per second of the line-cut profiles for the "control" and PC device.

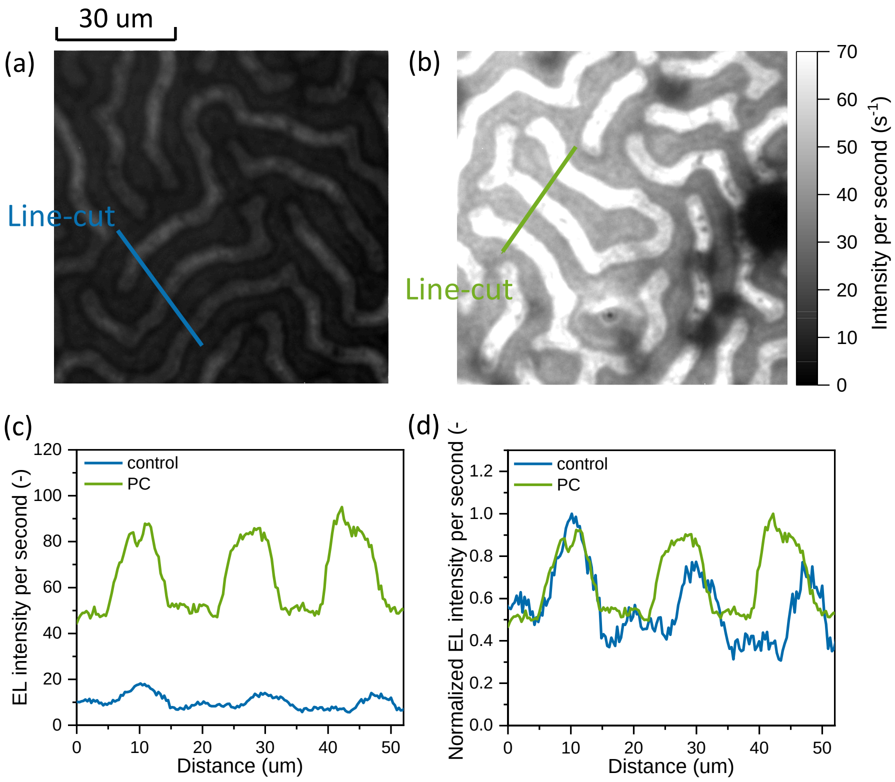

Fig. S21. The electroluminescence (EL) image of the (a) "control" and (b) PC device. EL images were obtained from the glass side. Both images have the same area and share the same scale bar. (c) The line-cut profile of the EL intensity per second extracted from the "control" (blue) and PC device (green). (d) Normalized line-cut profile of the EL intensity per second. Normalization is done by dividing the values to the maximum value.

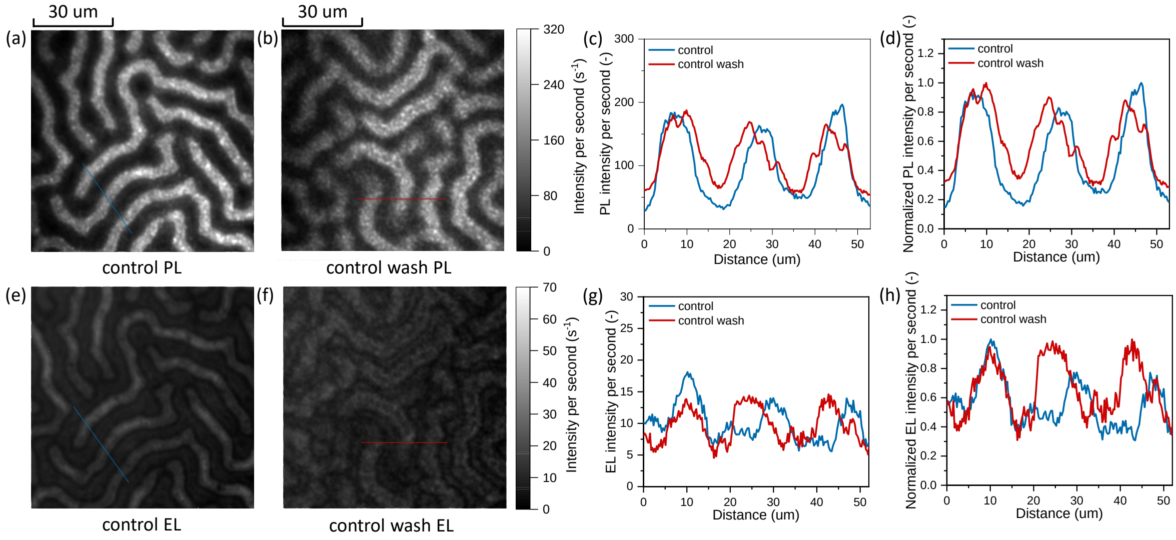

Fig. S22. The PL images of (a) "control" and (b) "control wash" device with the same length scale and scale bar. (c) The line-cut profiles and (d) normalized line-cut profiles of the "control" (blue) and "control wash" device (red) extracted from the corresponding PL images. The EL images of (e) "control" and (f) "control wash" device with the same length scale and scale bar. (g) The line-cut profiles and (h) normalized line-cut profiles of the "control" (blue) and "control wash" device (red) extracted from the corresponding EL images. The results of "control" and "control wash" devices are comparable.

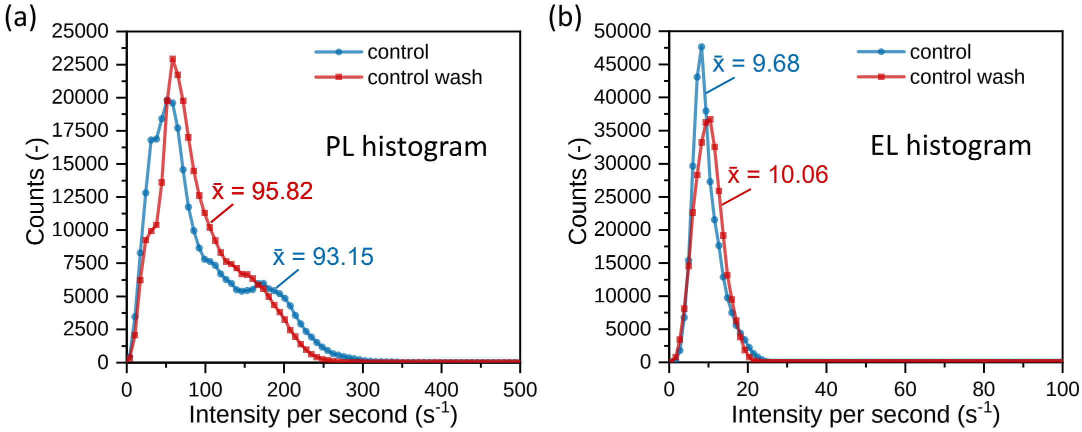

Fig. S23. (a) PL and (b) EL histograms extracted from the corresponding PL and EL images of the "control" and "control wash" device.

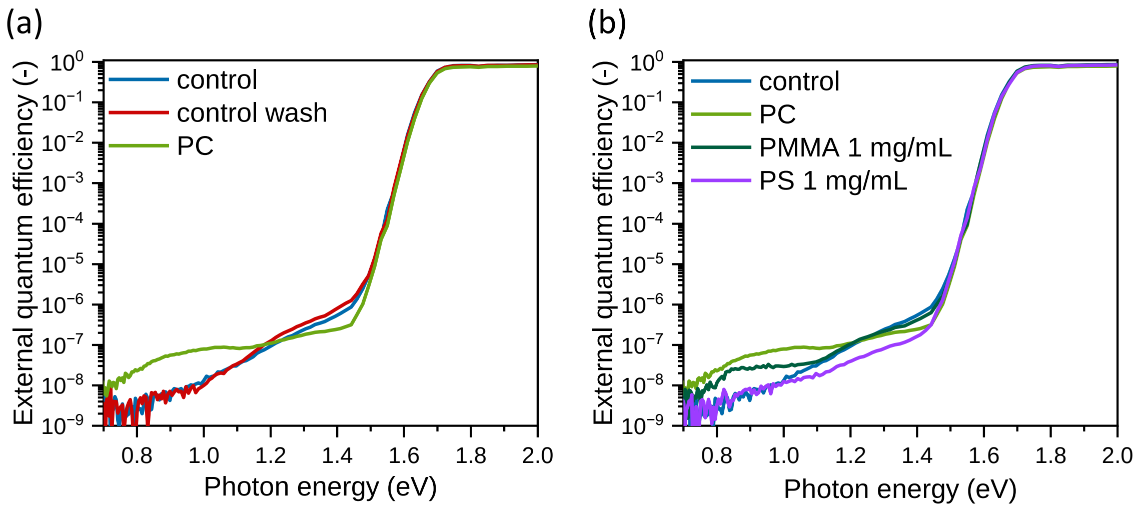

Fig. S24. (a) Sensitive EQE_{PV} spectra of "control", "control wash" and PC devices. (b) Sensitive EQE_{PV} spectra of "control", PC, PMMA thin layer passivated and PS thin layer passivated devices.

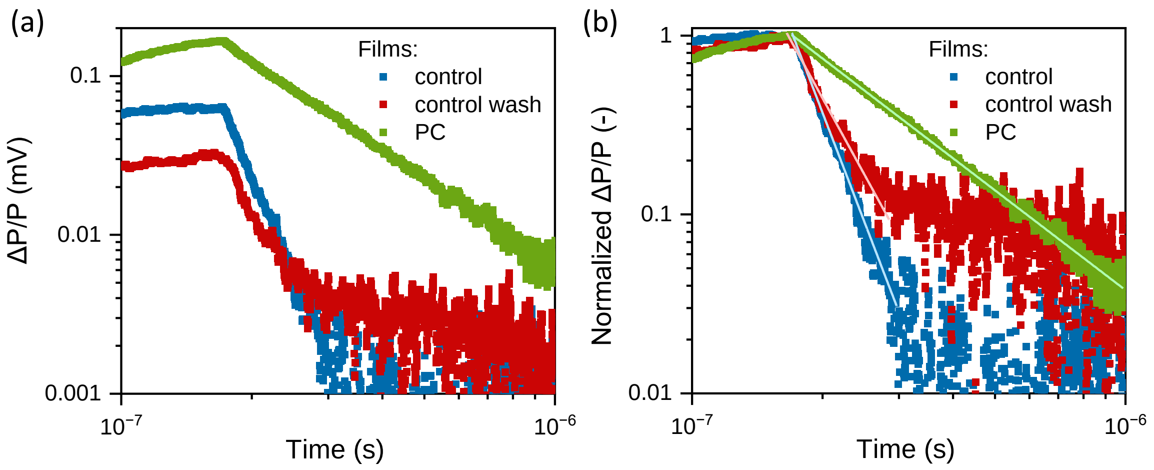

Fig. S25. (a) Time-resolved microwave conductivity (TRMC) transients and (b) normalized TRMC transients with the change in the reflected microwave power (ΔP/P) versus time (t) of the "control", "control wash" and PC perovskite films on glass substrate. The light green, light red and light blue curves in panel b are the fitting results of the decaying signals of "control", "control wash" and PC, respectively, via a power law decay function of $\Delta P/P = A \times t^{-\alpha}$, where A is a fitting constant and the extracted decay parameters (α) are 6.07, 4.45 and 1.85 for "control", "control wash" and PC, respectively. Note that we fit the decay of "control" and "control wash" in the first ~ 100 ns, since the noise will dominate afterwards. α is a fit parameter we use to quantify the decay behavior when a power-law dependence between time and TRMC value is observed.

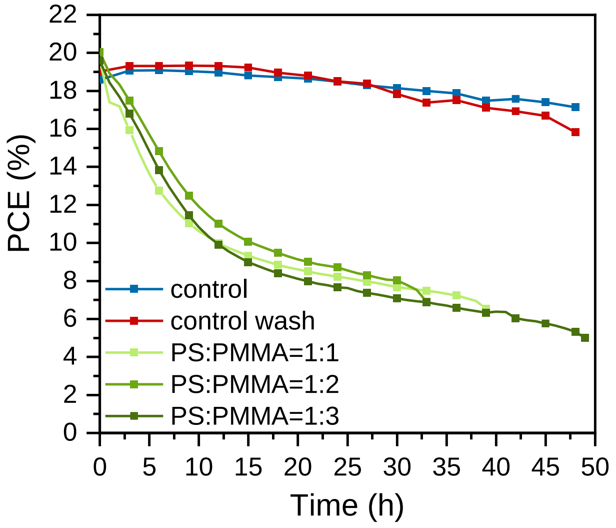

Fig. S26. Stability measurements on the "control", "control wash", PS:PMMA=1:1, PC (PS:PMMA=1:2) and PS:PMMA=1:3 device. The encapsulated devices are kept at maximum output voltage at 35 °C in air under white LED one-sun illumination and a *J-V* curve is measured with a reverse scan at a certain time interval.

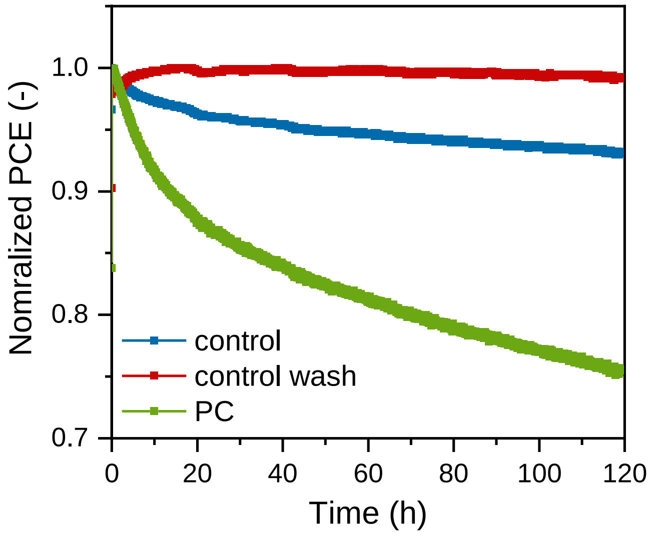

Fig. 27. Stability measurements on the "control", "control wash", PC (PS:PMMA=1:2) device. The devices are kept at maximum power output at 25 °C under nitrogen inside a glovebox under white LED illumination at one-sun equivalent intensity.

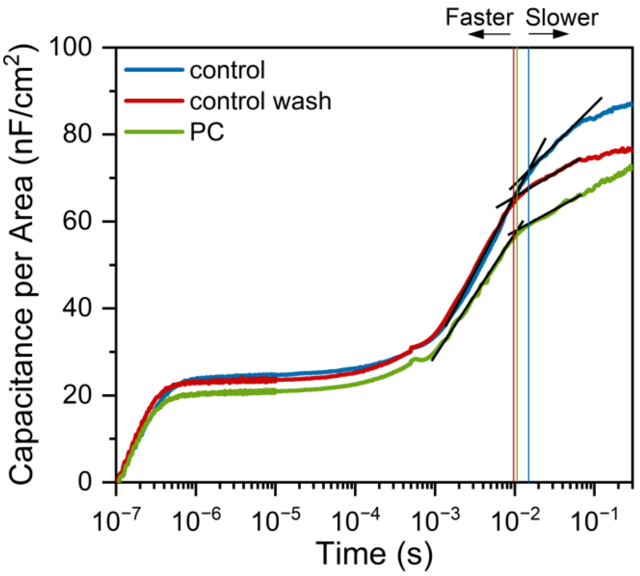

Fig. S28. The capacitance density of the devices versus time upon linearly increasing the applied voltage 0 V to -0.4 V.[7] The product of the capacitance per area with the maximum applied voltage (0.4 V in this case) at the time < ~ 10 μs estimates the electrode charge. The rise of the signal at ~ 0.1 ms corresponds to the ionic charge. According to $n_{ics} = C \times V/d/A$, where C is the capacitance; V is the applied voltage and equals 0.4 V in this case; d is the thickness of the active area and a value of 400 nm is used, A is the area of the device and equals 0.0648 cm^2. The ionic charge density (n_{ics}) can be calculated as 5.42×10^{15} cm^{-3}, 4.76×10^{15} cm^{-3} and 4.51×10^{15} cm^{-3} for the "control", "control wash" and PC device, respectively, indicating comparable mobile ion density. See Experimental Section and Ref [1] for more details. Faster and slower mobile ions can be distinguished with the extrapolation of the corresponding curves. The slower mobile ion of the "control", "control wash" tends to saturate at a longer time, while that of the PC device keeps increasing.

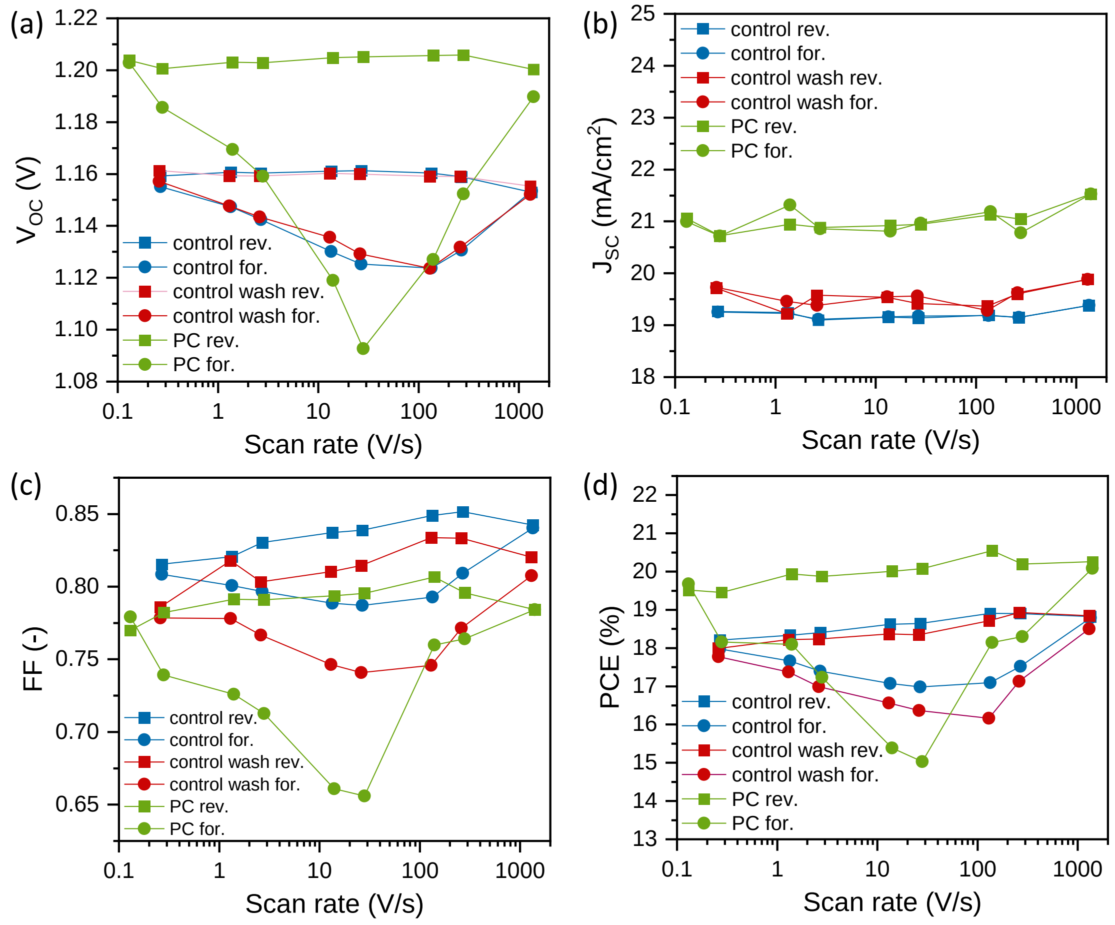

Fig. S29. (a) V_{OC}, (b) J_{SC}, (c) FF, and (d) PCE of the "control" "control wash" and PC device measured with forward and reverse scan at a scan rate ranging from ~ 0.2 to ~ 1400 V s^{-1}.

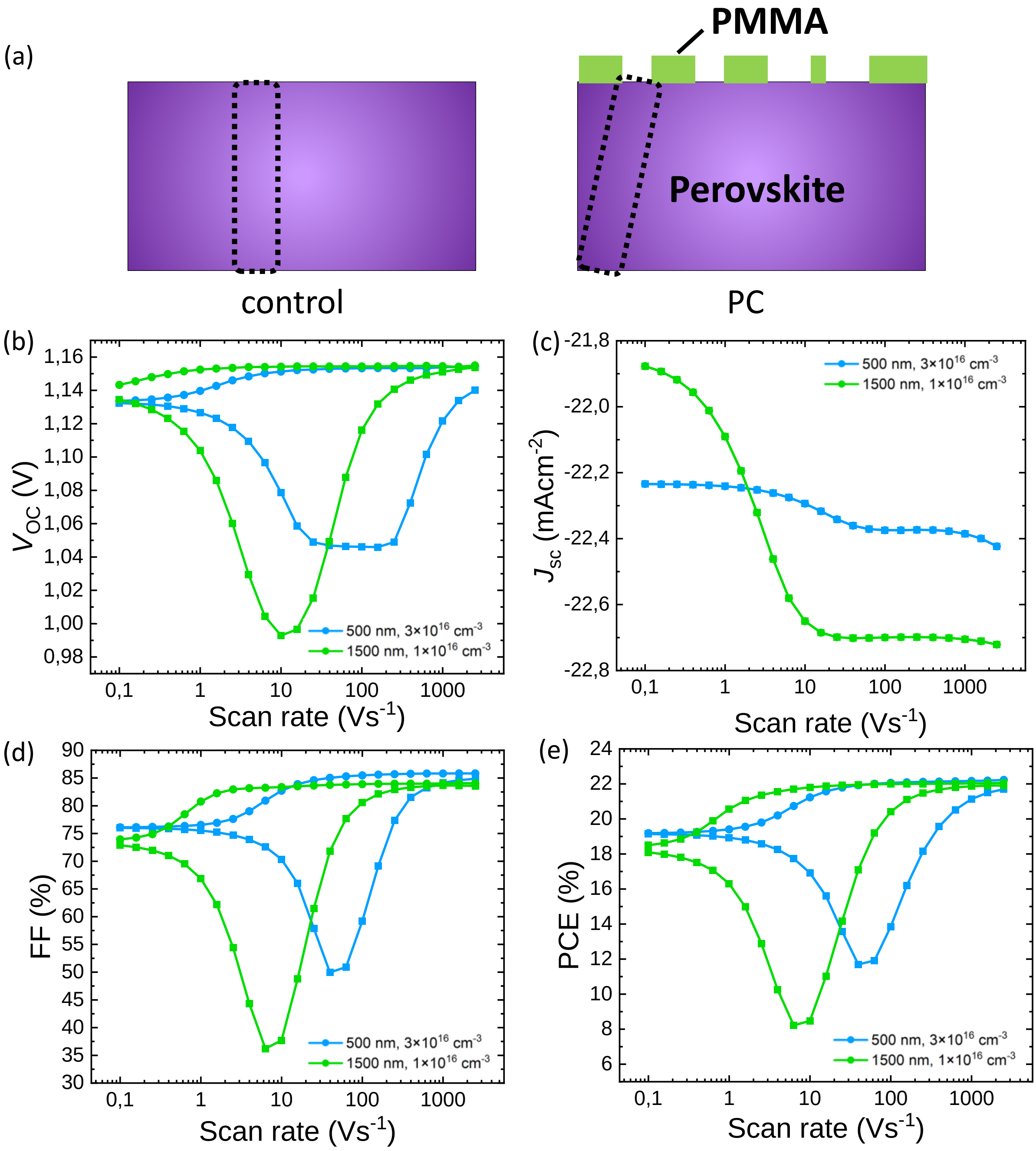

Fig. S30. (a) A schematic figure showing that the percolation length of the charge carriers and the distance of mobile ion migration are increased with the PC strategy. Based on this, we perform a simulation on fast hysteresis (FH) measurements by increasing the thickness of the perovskite layer by a factor of 3, and meanwhile decreasing the ion density by the same factor to keep the same mobile ion numbers. The (b) V_{OC}, (c) J_{SC}, (d) FF and (e) PCE changes according to the scan rate of the FH measurements simulation. The FH simulation confirms the hysteresis peak shift in V_{OC}, FF and PCE from a high scan rate to a lower one for the PC device observed in Fig. 3d in the main text and Fig. S29.

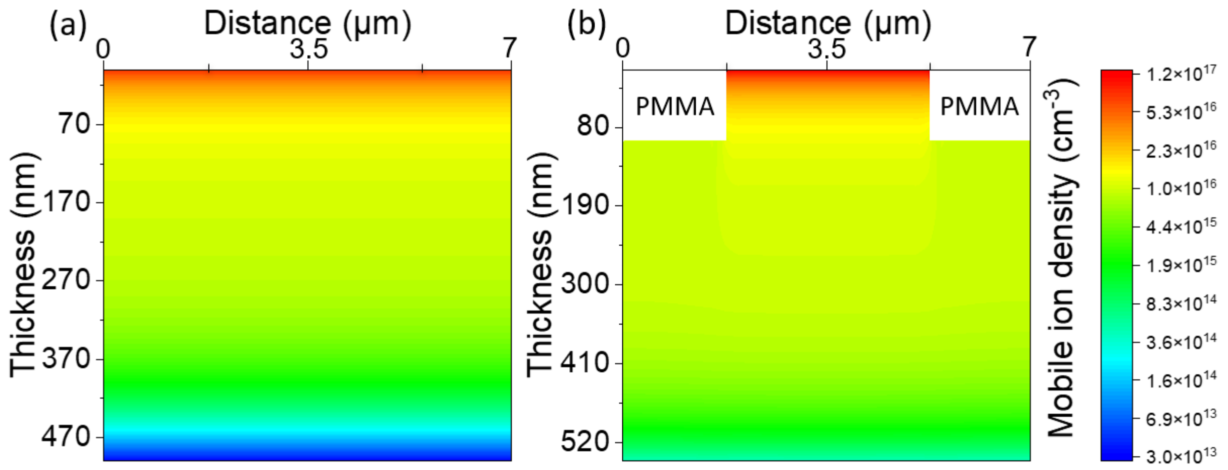

Fig. S31. The simulated distribution of the mobile ions of the (a) "control" and (b) PC perovskite layer at steady state under an applied voltage (V_{app}) of 0.95 V with an overall mobile ion density of 1 × 10^{16} cm^{-3}. Note that the range of the color scale bar is larger, compared to that in the main text. The thickness in the PC perovskite is increased to ensure the same volume as the "control". The simulation details are provided in Simulation S2 in the ESI†.

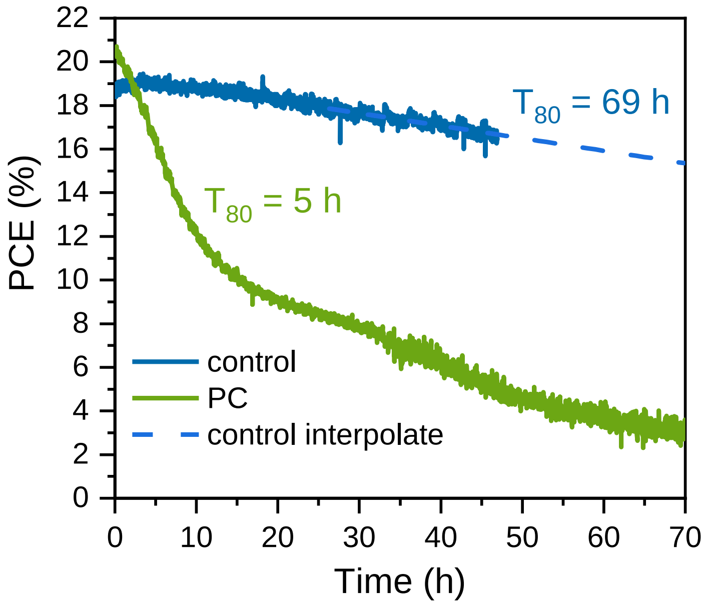

Fig. S32. The Stability measurements on the "control", and PC device. The encapsulated devices are held at 35 °C in ambient air under white LED one-sun equivalent illumination and kept at a maximum power output voltage. To get the time, when the "control" device reaches 80% of its initial PCE (T_{80}), an interpolation is used. The T_{80} is 69 h for the "control" and 5 h for the PC device.

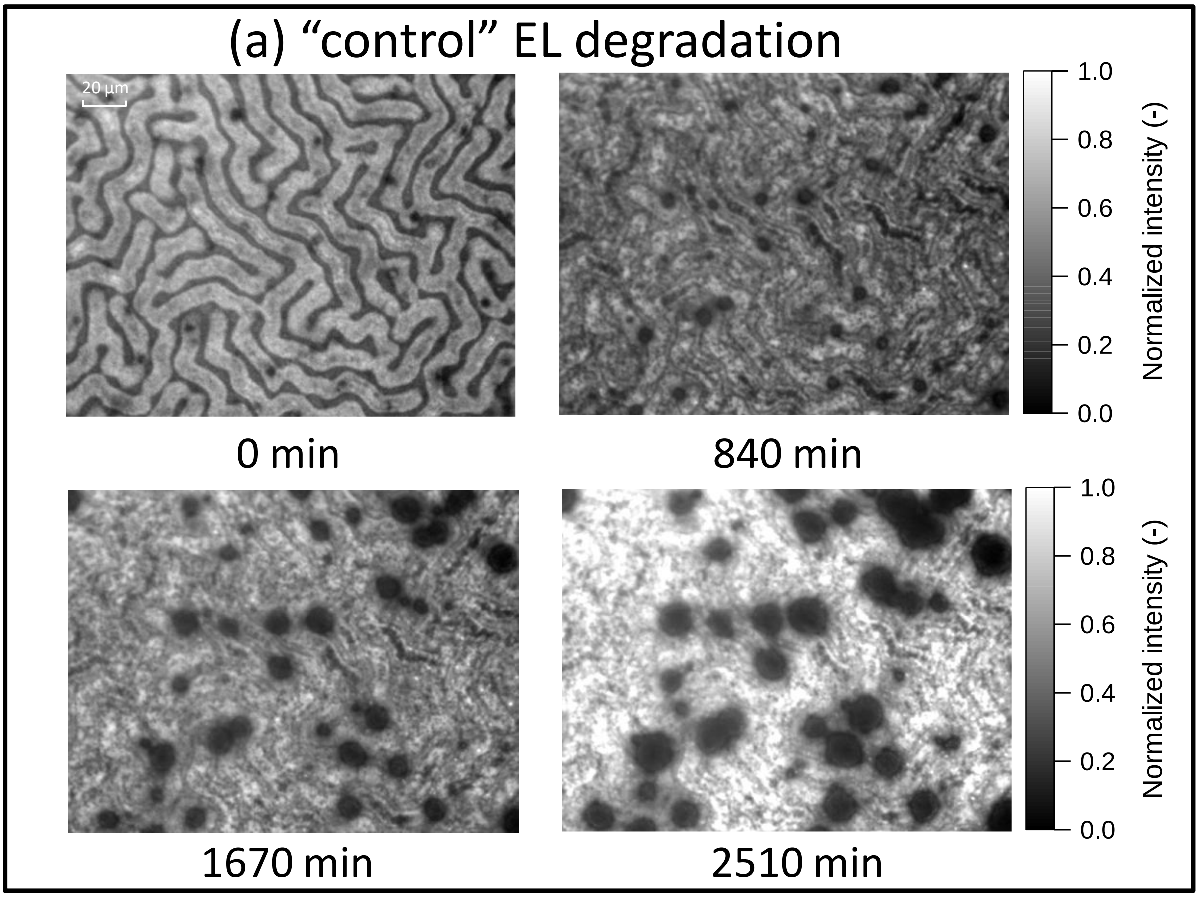
(a) "control" EL degradation
20 µm
0 min
840 min
1670 min
2510 min
Normalized intensity (-)
1.0
0.8
0.6
0.4
0.2
0.0

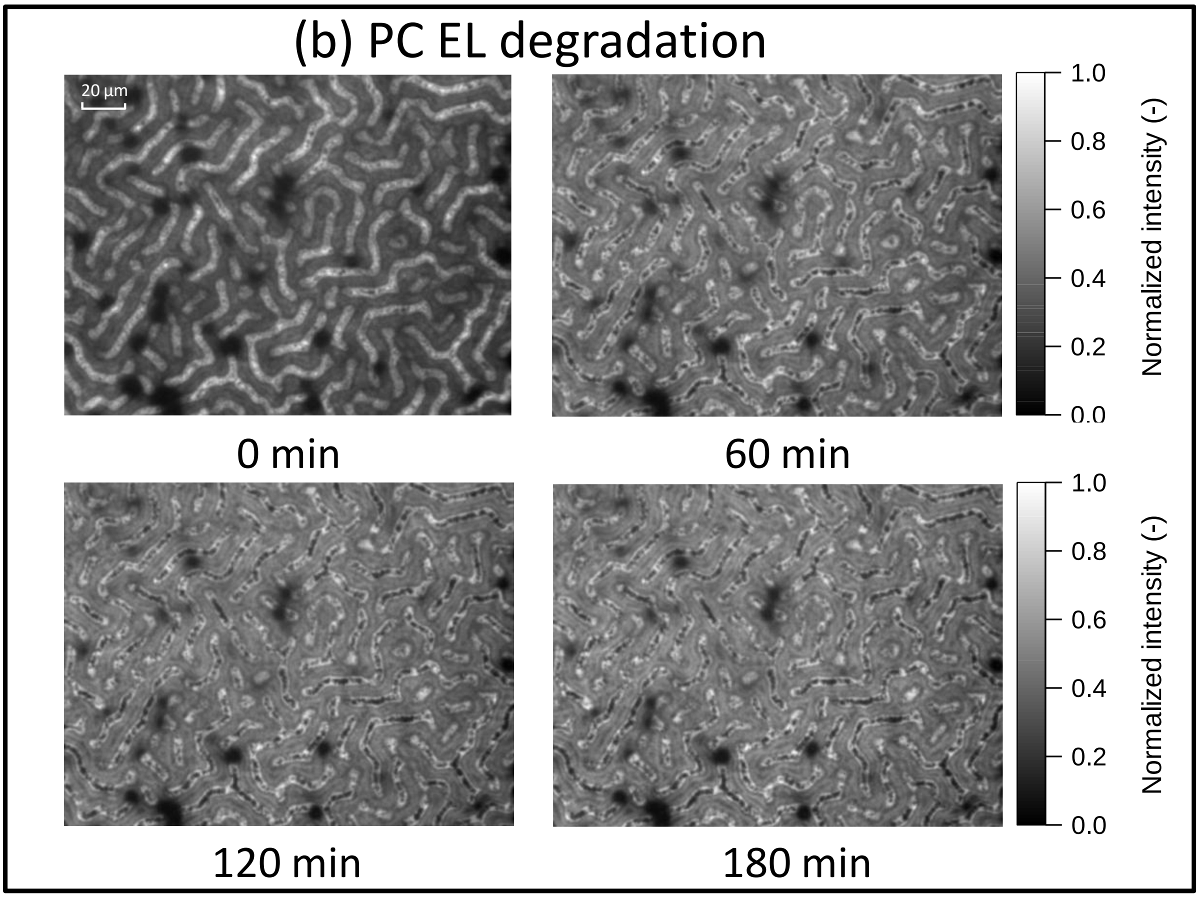
(b) PC EL degradation
20 µm
0 min
60 min
120 min
180 min
Normalized intensity (-)
1.0
0.8
0.6
0.4
0.2
0.0

Fig. 33. Normalized in-situ EL imaging of the (a) "control" and (b) PC device. The devices are encapsulated and kept at a five-sun-equivalent injection current in the ambient air. Every 10 minutes, an EL image at the same spot was taken. To ensure a comparable degradation in the PCE, we compare the EL images at different times for the "control" and PC device (840 min versus 60 min, 1670 min versus 120 min and 2510 min versus 180 min), according to the ratio of T_{80} (13.8) in Fig. S32. Data are normalized by dividing to the maximum value. Videos are provided in Video S1-S2 in the ESI†.

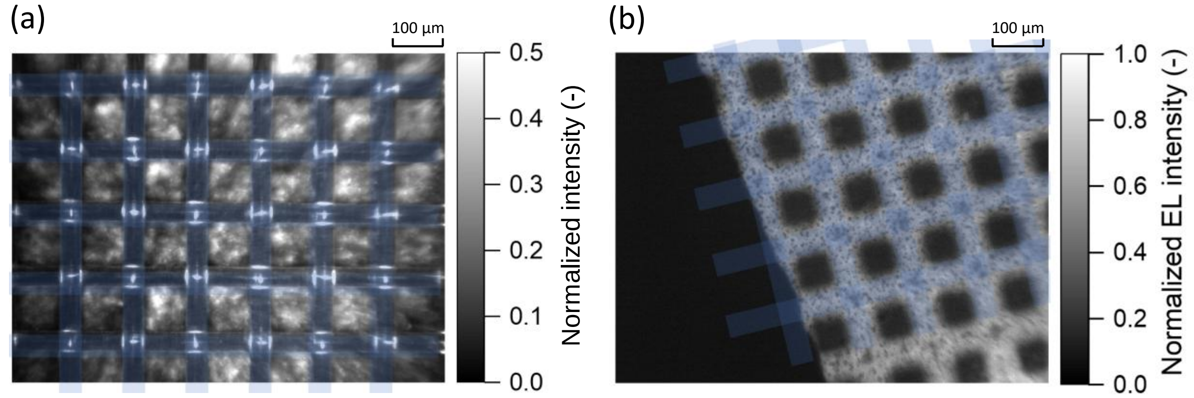

Fig. S34. (a) Nylon mesh on a metal platform under an optical microscope. A blue shaded pattern is overlaid on top. The nylon mesh is used as an evaporation mask to form LiF patterns. (b) EL images of mesh PC (M-PC) perovskite device based on $Cs_{0.05}FA_{0.98}MA_{0.02}Pb(I_{0.98}Br_{0.02})_3$ recipe, which does not show the wrinkled structures. The same blue shaded pattern in panel (a) is overlaid on top to demonstrate that the brighter patterns are the point contacts and the darker rectangles are covered with LiF.

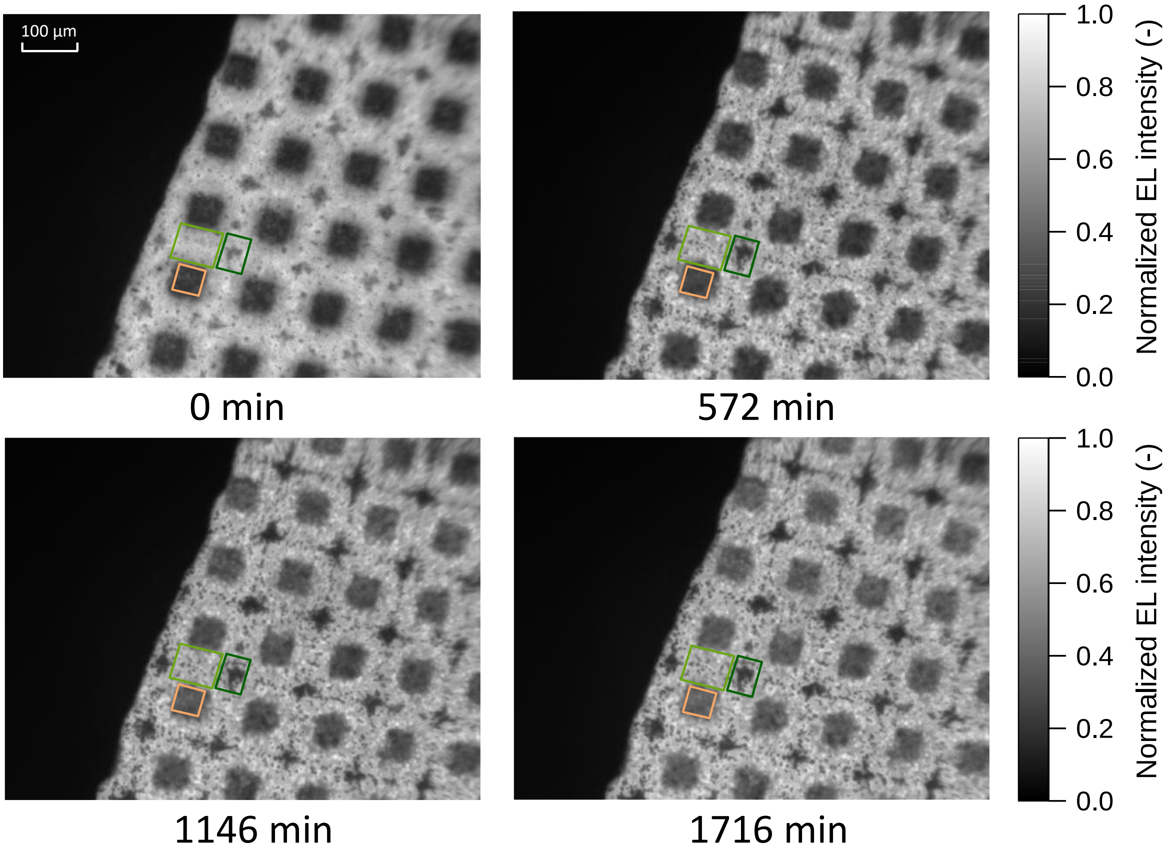

Fig. S35. Normalized in-situ EL imaging of the mesh PC device based on $Cs_{0.05}FA_{0.98}MA_{0.02}Pb(I_{0.98}Br_{0.02})_3$ recipe. The devices are encapsulated and kept at a two-sun-equivalent injection current in the ambient air. Every two minutes, an EL image at the same spot was taken. Data are normalized by dividing to the maximum value. The video is provided in Video S3 in the ESI†.

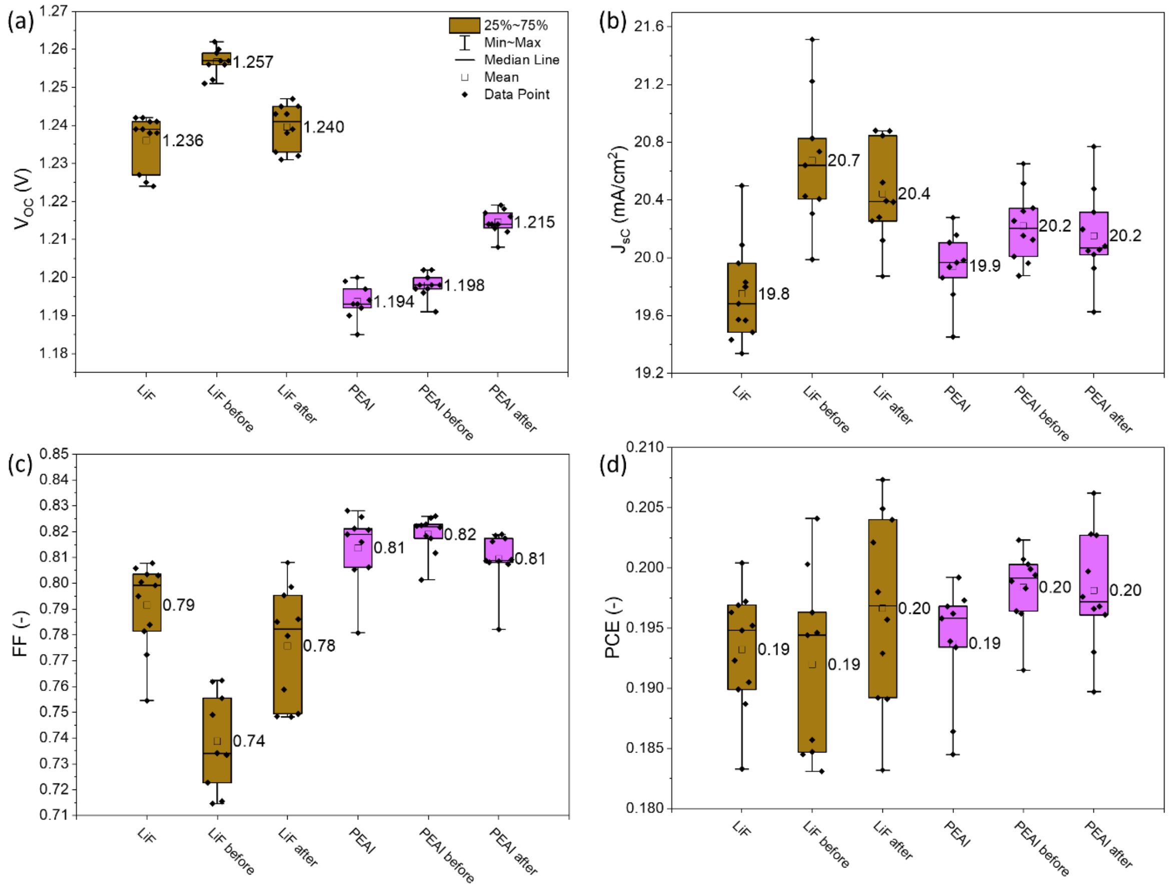

Fig. S36. The box diagram of (a) V_{OC}, (b) J_{SC}, (c) FF and (d) PCE of LiF or PEAI passivated, LiF or PEAI before or after PC devices under AM 1.5G, 100 mW cm^{-2} illumination with scatters of data points.

Supplementary Table

Table S1. The measured photoluminescence quantum yield (PLQY) and quasi-Fermi level splitting (QFLS) of the samples based on film and device structure with different conditions.

Devices and films	PLQY (-)	QFLS (eV)	V_{OC} (V)	J_{SC}[a)] (A/m^2)	$J_{0,\ rad}$[a)] (A/m^2)
control	1.94E-04	1.162	1.16	200	9.06E-22
control wash	2.30E-04	1.168	1.16	199.5	8.48E-22
PS:PMMA=1:1	1.02E-03	1.203	1.20	204	9.75E-22
PS:PMMA=1:2	1.55E-03	1.215	1.21	210	9.75E-22
PS:PMMA=1:3	9.23E-04	1.201	1.21	206	9.75E-22
Glass/Pero	0.0155	1.274	/	200	9.06E-22
Glass/Pero/PS:PMMA=1:2	0.00646	1.252	/	200	9.06E-22

[a)] For PS:PMMA=1:1 and PS:PMMA=1:3 devices, the same $J_{0,\ rad}$ as PS:PMMA=1:2 is used for PLQY calculation. For the film structure, the same J_{SC} and $J_{0,\ rad}$ as the "control" device is used.

Table S2. The photovoltaic parameters of the simulated "control" and PC devices with varied mobile ion density.

Mobile ion density (cm^{-3})	1×10^{18}		1×10^{16}		0	
Device	control	PC	control	PC	control	PC
J_{SC} (mA/cm^2)	18.69	18.84	19.96	19.99	20.01	20.02
V_{OC} (V)	0.99	1.03	1.04	1.04	1.09	1.11
FF (-)	0.660	0.638	0.809	0.790	0.869	0.874
PCE (%)	12.2	12.4	16.8	16.4	19.0	19.4

1. V. M. Le Corre, J. Diekmann, F. Peña-Camargo, J. Thiesbrummel, N. Tokmoldin, E. Gutierrez-Partida, K. P. Peters, L. Perdigón-Toro, M. H. Futscher, F. Lang, J. Warby, H. J. Snaith, D. Neher and M. Stolterfoht, *Solar RRL*, 2022, **6**, 2100772.
2. M. Stolterfoht, M. Grischek, P. Caprioglio, C. M. Wolff, E. Gutierrez-Partida, F. Peña-Camargo, D. Rothhardt, S. Zhang, M. Raoufi, J. Wolansky, M. Abdi-Jalebi, S. D. Stranks, S. Albrecht, T. Kirchartz and D. Neher, *Advanced Materials*, 2020, **32**, 2000080.
3. P. Wurfel, *Journal of Physics C: Solid State Physics*, 1982, **15**, 3967.
4. W. Shockley and H. J. Queisser, *Journal of Applied Physics*, 1961, **32**, 510-519.
5. D. A. Jacobs, C. M. Wolff, X.-Y. Chin, K. Artuk, C. Ballif and Q. Jeangros, *Energy & Environmental Science*, 2022, **15**, 5324-5339.
6. B. Roose, K. Dey, Y.-H. Chiang, R. H. Friend and S. D. Stranks, *The Journal of Physical Chemistry Letters*, 2020, **11**, 6505-6512.
7. G. Juška, K. Arlauskas, M. Viliūnas, K. Genevičius, R. Österbacka and H. Stubb, *Physical Review B*, 2000, **62**, R16235-R16238.
